# Role of Oxygen Defects in Eliciting a Divergent Fluorescence Response of Single-Walled Carbon Nanotubes to Dopamine and Serotonin

**DOI:** 10.1021/acsnano.4c10360

**Published:** 2024-12-05

**Authors:** Srestha Basu, Adi Hendler-Neumark, Gili Bisker

**Affiliations:** † Department of Biomedical Engineering, Faculty of Engineering, Tel Aviv University, Tel Aviv 6997801, Israel; ‡ Center for Physics and Chemistry of Living Systems, Tel Aviv University, Tel Aviv 6997801, Israel; § Center for Nanoscience and Nanotechnology, Tel Aviv University, Tel Aviv 6997801, Israel; ∥ Center for Light-Matter Interaction, Tel Aviv University, Tel Aviv 6997801, Israel

**Keywords:** single-walled carbon nanotubes, defects, neurotransmitters, fluorescence, dopamine, serotonin

## Abstract

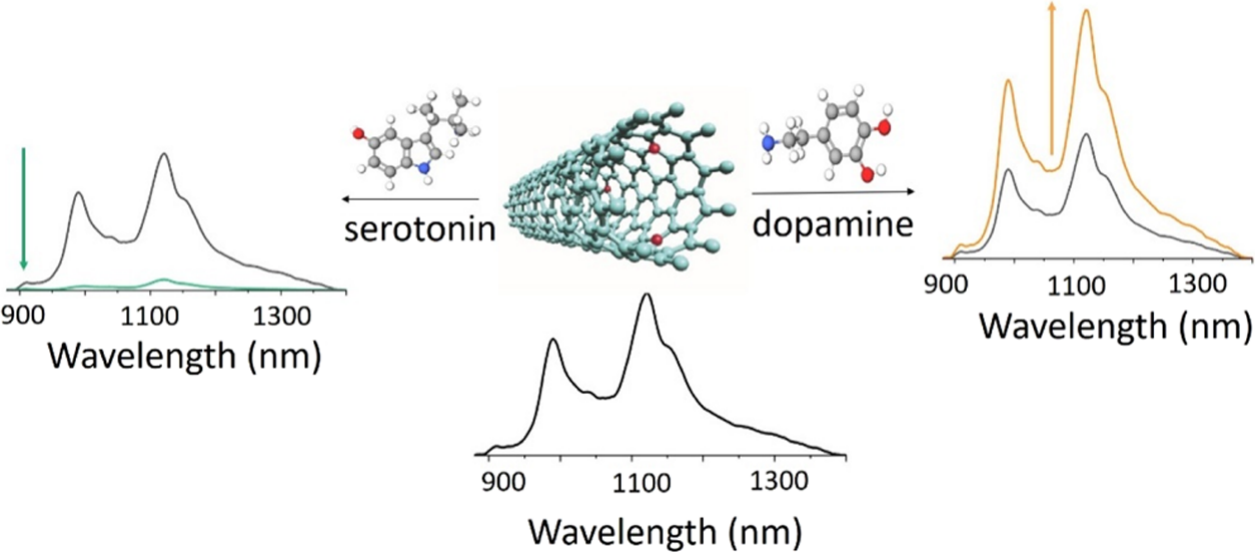

Modulating the optical response of fluorescent nanoparticles through rational modification of their surface chemistry can yield distinct optical signatures upon the interaction with structurally related molecules. Herein, we present a method for tuning the fluorescence response of single-walled carbon nanotubes (SWCNTs) toward dopamine (DA) and serotonin, two structurally related monoamine-hydroxylated aromatic neurotransmitters, by introducing oxygen defects into (6,5) chirality-enriched SWCNTs suspended by sodium cholate (SC). This modification facilitated opposite optical responses toward these neurotransmitters, where DA distinctly increased the fluorescence of the defect-induced emission of SWCNTs (D-SWCNTs) 6-fold, while serotonin notably decreased it. In contrast, pristine, defect-free SWCNTs exhibited similar optical responses to both neurotransmitters. The underlying mechanisms for the divergent fluorescence response were found to be polydopamine (PDA) surface adsorption in the case of the fluorescence enhancement in response to DA, while the fluorescence decrease in response to serotonin was attributed to enhanced solvent relaxation effects in the presence of defects. Importantly, the divergent optical response of D-SWCNTs to DA and serotonin, via the introduction of defects, was validated in complex biological environments such as serum. Further, the generality of our approach was confirmed by the demonstrations of a divergent fluorescence response of D-SWCNTs suspended by an additional dispersant, namely lipid–polyethylene glycol (PEG). This study offers promising avenues for the broad applicability of surface functionalization of SWCNTs to achieve divergent responses toward structurally related molecules and advance applications in sensing, imaging, and diagnostic technologies.

## 1 Introduction

The interaction of optical probes with exogenously added molecules is primarily dictated by the chemical structure of these molecules, resulting in alterations in the optical signatures of the probes, thereby enabling the successful detection of the target molecules.^1,2^ Therefore, molecules with related structural features often elicit similar optical responses from the probes, making their clear-cut distinction a significant challenge.^3,4^ Thus, the development of techniques for tailored functionalization of optical probes that give rise to divergent optical responses toward structurally analogous analytes is a challenge worthy of pursuit.

To this end, single-walled carbon nanotubes (SWCNTs), which exhibit fluorescence in the beneficial near-infrared (NIR) region and feature noteworthy photostability, ease of surface functionalization, biocompatibility, tunable surface chemistry, and fluorescence modulation following analyte binding and surface composition, have emerged as optical probes capable of responding to a wide array of analytes.^5−9^ These include proteins,^10−12^ lipids,^13,14^ sugars,^15−17^ hormones, metal ions,^18^ oncometabolites,^19^ volatiles,^20,21^ pathogens,^22,23^ micro-RNA,^24,25^ neurotransmitters,^26−30^ enzyme activity, and inhibition,^31−33^ among others. Furthermore, SWCNTs are known for their well-defined graphene-like surfaces, which largely govern their interactions with exogenously added molecules. Thus, tailoring the surface of SWCNTs with functional modalities presents an interesting and realistic approach to tuning their surface chemistry. This, in turn, enables the customization of their responses to molecule libraries sharing common features that would otherwise evoke similar optical responses of the SWCNTs.

In this context, a promising technique for introducing additional functionalities into SWCNTs involves the incorporation of sp^3^ defects.^34−45^ These deliberately introduced defects not only lead to the emergence of intriguing optical properties but also provide an additional platform for selective interaction with chosen analytes, thereby allowing for selective molecular recognition. In this regard, aryl defects-incorporated SWNCTs have been used as sensors for the detection of a number of analytes.^46−49^ However, the utility of exogenously incorporated defects in SWCNTs for modulating their optical response toward a particular analyte is rather limited in the literature. Furthermore, achieving divergent optical responses to two structurally related molecules, thereby leading to their successful discrimination, could be a direct consequence of the surface functionalization of SWCNTs with defects. This approach not only holds significant promise for advancing the field of selective molecular recognition using SWCNTs but also lays the groundwork for deterministic modulation of the surface chemistry of SWCNTs to achieve tailored optical responses.

Among a wide variety of structurally related molecules, neurotransmitters stand out as particularly important due to their similar functional groups and parallel structural frameworks.^50,51^ Despite these structural similarities, each neurotransmitter exerts distinct effects on the neurological system. For instance, DA and serotonin are both monoamine-based neurotransmitters that share hydroxyl groups and aromatic rings. However, their impacts on the neurological system differ significantly. Given their structural similarities, it would be straightforward to expect similar optical responses to any optical probe, such as NIR fluorescent SWCNTs. Previous reports demonstrated the real-time imaging of serotonin release from human blood platelets using SWCNTs functionalized by serotonin-binding DNA aptamer.^52^ Moreover, SWCNTs suspended with various single-stranded DNA (ssDNA) sequences were employed for DA detection and imaging.^53,54^ Additionally, other varieties of chirality-pure SWCNTs were also employed for DA sensing.^55^ Using a different approach, the “systematic evolution of ligands by exponential enrichment implemented on SWCNT surfaces” – termed SELEC – was established as an efficient method for high-throughput screening of ssDNA to achieve sensitive detection of serotonin.^30^ Furthermore, sp^3^ defects functionalized SWCNTs were utilized for ratiometric imaging of catecholamine neurotransmitters.^3^ Nevertheless, the divergent impacts of DA and serotonin on health, behavior, and diseases emphasize the importance of developing techniques that use the principles of chemistry to alter the surface functionalization of SWCNTs and enable contrasting optical responses of the SWCNT sensors to these structurally related neurotransmitters. Achieving such divergent effects from structurally close molecules, by virtue of the introduction of oxygen defects in SWCNTs, holds great potential not only for advancing our understanding of the chemical behavior of these neurotransmitters, which play vital roles in neurological function and disorders but also for the development of tools to allow tailored optical responses from optical probes.

**Scheme 1:**
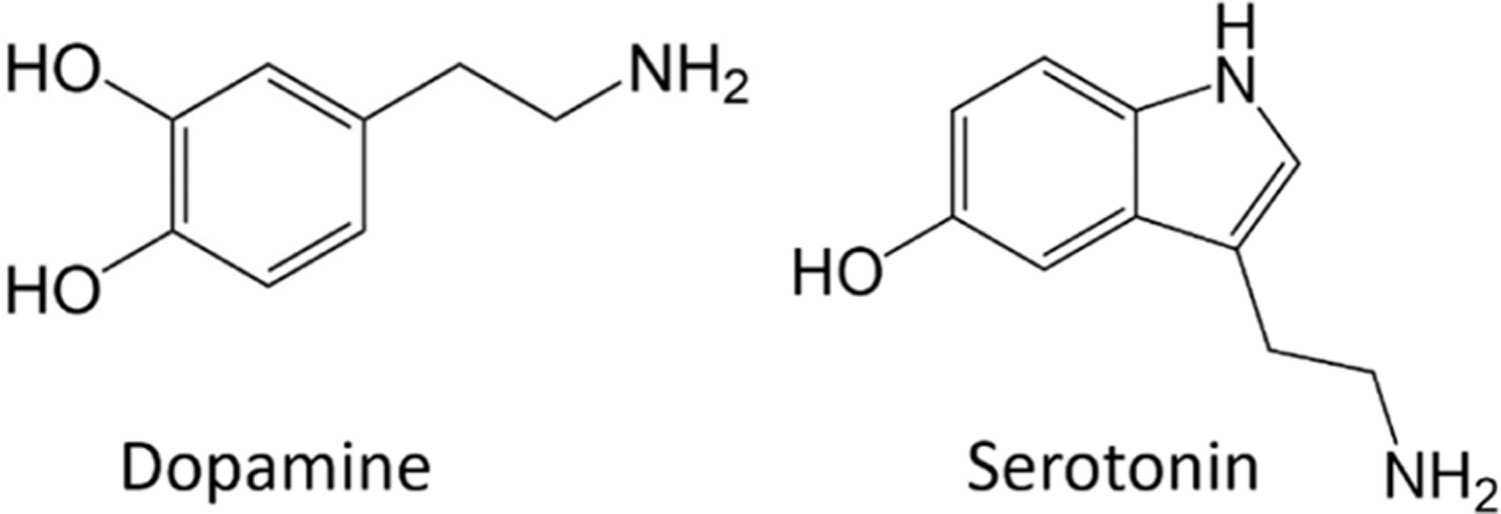
Molecular Structures of DA and Serotonin

Herein, we report an approach of introducing substitutional defects in SWCNTs to achieve distinctive fluorescence responses when exposed to DA and serotonin (Scheme 1), which otherwise evoked allied optical responses from SWCNTs without defects. To this end, oxygen defects were introduced into sodium cholate (SC) suspended SWCNTs via reaction with sodium hypochlorite (NaClO) under UV irradiation. UV–vis-NIR absorbance, NIR fluorescence, and Raman spectroscopic studies verified the successful incorporation of the oxygen defects in the SC-SWCNTs. DA and serotonin, despite sharing the characteristic of being monoamine-hydroxylated aromatic neurotransmitters, demonstrated divergent effects on the fluorescence of the D-SWCNTs. Specifically, DA caused a notable 6-fold increase in the fluorescence intensity of the D-SWCNTs, while serotonin resulted in a discernible intensity decrease. Notably, the distinction between DA and serotonin was minimal when pristine SWCNTs without defects were employed. The amount of defects introduced into the SWCNTs, characterized by the E_11_ to E_11_* peak ratio, played a critical role in distinguishing DA and serotonin. Explorations into the mechanisms through Raman spectroscopy and electrospray ionization mass spectrometry unveiled that the enhancement in the fluorescence intensity of D-SWCNTs when exposed to DA could be attributed to the surface adsorption of D-SWCNTs by polydopamine (PDA). In contrast, the distinct decrease in the fluorescence intensity of D-SWCNTs upon exposure to serotonin could be attributed to enhanced solvent relaxation of the SWCNTs in the presence of oxygen defects. Moreover, the effectiveness of defects in generating contrasting optical responses SWCNTs to DA and serotonin has been demonstrated in complex biological environments, such as fetal bovine serum (FBS). Furthermore, the generality of the principle of using oxygen defects to modulate the optical responses of SWCNTs toward DA and serotonin has been validated with defect-induced emission of SWCNTs suspended by Polyethylene glycol (PEG)-lipid corona. Our findings not only mark an important instance of DA and serotonin optical differentiation using defect-incorporated SWCNTs but also offer promising avenues for surface functionalization of SWCNTs for advancing their innovative applications in sensing, imaging, and diagnostic technologies.

## 2 Results and Discussion

### 2.1 Incorporation of Oxygen Defects into Sodium Cholate (SC) SWCNTs

**Figure 1:**
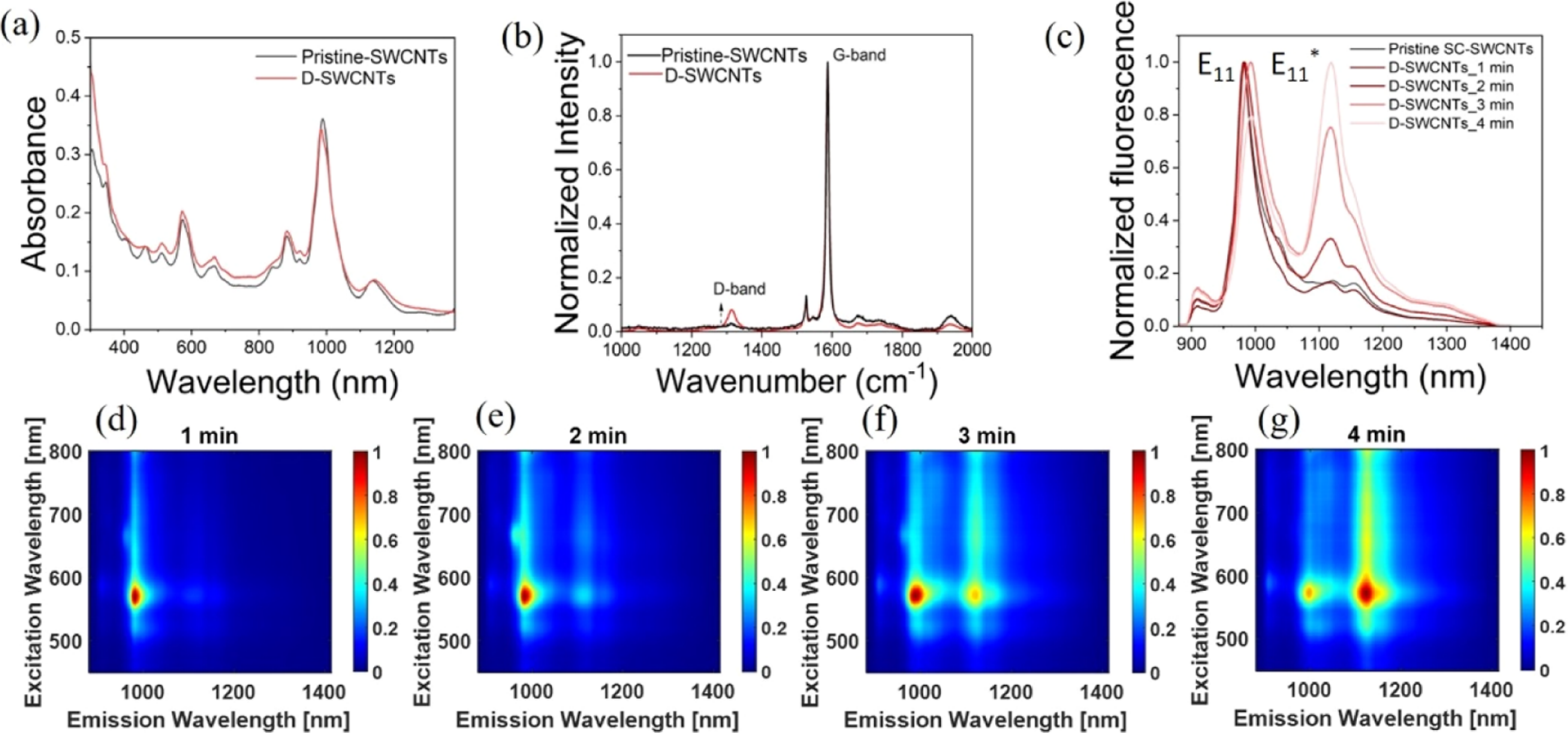
(a) UV–vis-NIR absorption spectra of pristine SC-SWCNTs (black curve) and D-SWCNTs (red curve). (b) Raman spectra of pristine SC-SWCNTs (black curve) and D-SWCNTs (red curve). (c) Normalized fluorescence spectra of SC-SWCNTs upon addition of NaClO with increasing duration of UV irradiation. Excitation–emission map of D-SWCNTs with UV exposure for (d) 1 min, (e) 2 min, (f) 3 min, and (g) 4 min.

The introduction of oxygen defects into (6,5) enriched SC-SWCNTs i.e., CoMoCAT nanotubes, was accomplished through a minor modification of a previously established procedure^56^ involving the reaction of controlled amounts of SC-SWCNTs and NaClO under UV irradiation. To assess the impact of oxygen defects, the UV–vis-NIR absorption spectrum of pristine SC-SWCNTs was compared to that of the D-SWCNTs (Figure 1a). In agreement with prior findings,^56^ the absorption peak associated with the E_11_ transitions in pristine SC-SWCNTs at 988 nm slightly decreased after incorporating oxygen defects as a result of the perturbations induced by the covalent modifications. It is important to note that the chemical bonding of oxygen defects within SWCNTs is reported to occur through the formation of ether or epoxide.^56−58^

Subsequently, Raman spectra of both pristine SC-SWCNTs and D-SWCNTs were obtained to confirm the successful inclusion of oxygen defects. Consistent with previous literature reports, the introduction of point-like defects in the otherwise sp^2^ carbon lattice increased the Raman D mode, when normalized against the G band. Accordingly, the Raman spectra of D-SWCNTs displayed the emergence of a D band at 1312 cm^–1^, alongside the G band at 1587 cm^–1^, validating the incorporation of oxygen defects into SC-SWCNTs (Figure 1b).^48,59^

The reaction time-dependent fluorescence spectra of SC-SWCNTs incubated with NaClO under UV irradiation were also monitored to assess the effect of the defects on the SWCNT fluorescence. The successful incorporation of oxygen defects was evidenced by the gradual appearance of a fluorescence peak at 1120 nm (E_11_*), in addition to the existing peak at 988 nm (E_11_). As evinced from the normalized fluorescence spectra of SC-SWCNTs before and after treatment with NaClO, with an increase in UV exposure from 1–4 min, the peak at 1120 nm exhibited a gradual augmentation (Figure 1c). Notably, the overall fluorescence of the SC-SWCNTs decreased following the incorporation of defects (Figure S1). This observation suggests that mobile excitons are captured at defect sites and recombine to emit fluorescence, resulting in a decrease in the emission from the E_11_ states. In conjunction, the corresponding excitation–emission profile of the D-SWCNT fluorescence was recorded as a function of increasing defect concentration. These profiles featured the progressive emergence of the fluorescence peak attributed to E_11_*, thus clearly confirming the successful introduction of oxygen defects (Figure 1d–g). An important observation from these results was that the number of defects in the D-SWCNTs could be regulated by controlling the duration of UV irradiation.

### 2.2 Modulating the Response of DA and Serotonin toward SWCNTs by the Introduction of Oxygen Defects

Having confirmed the successful incorporation of oxygen defects in chirality-enriched SC-SWCNTs, we then focused on evaluating their efficacy as probes for modulating the optical response of structurally related analytes with similar functional groups. Neurotransmitters, which typically consist of allied functional groups and thus result in similar optical responses of conventional probes, were chosen as model analytes. Our objective was to determine if the introduction of oxygen defects in SWCNTs could differentiate these analytes by altering their optical interactions with the SWCNTs.

**Figure 2:**
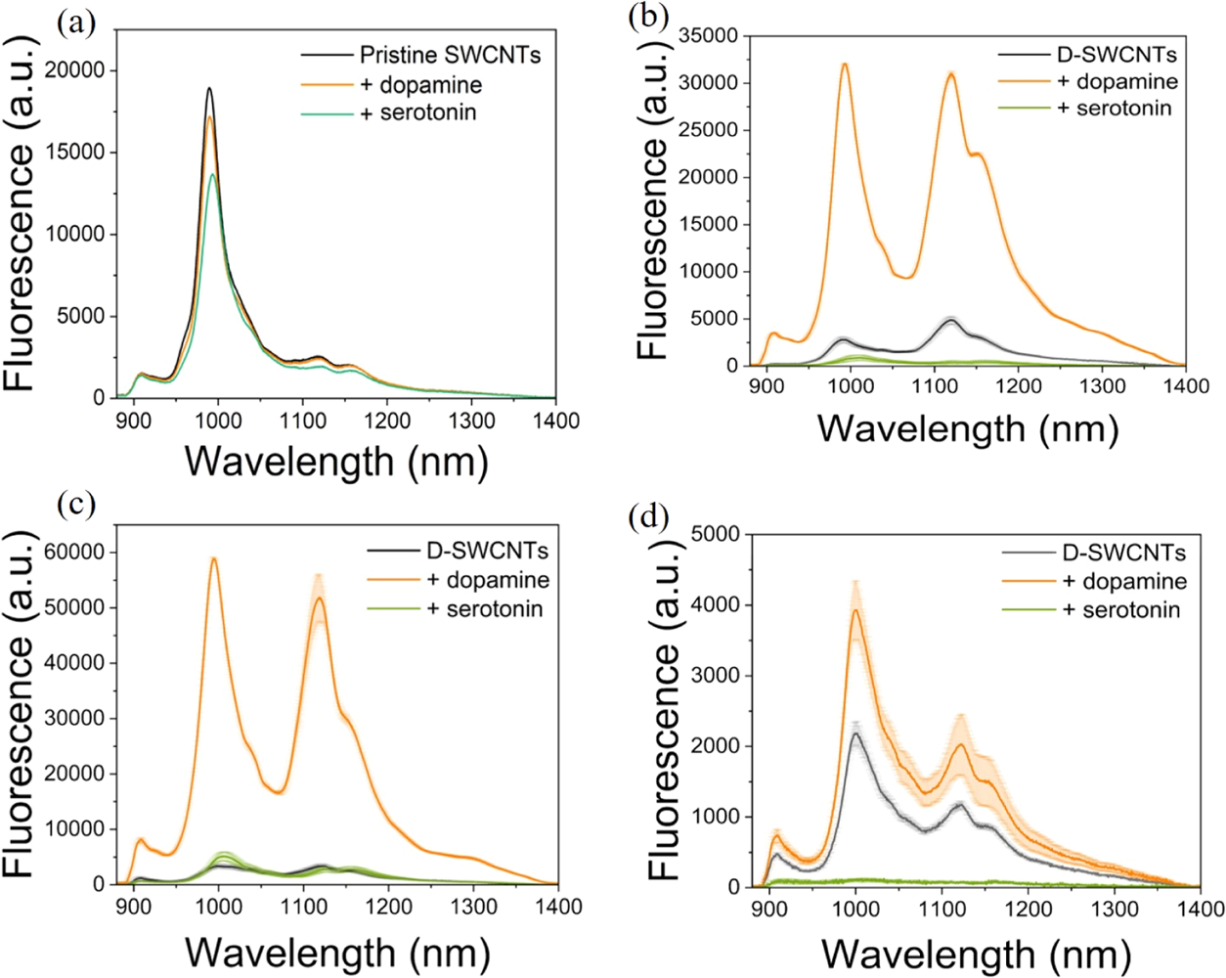
(a) Fluorescence spectra of pristine SC-SWCNTs (black curve) and that following the addition of dopamine (orange curve) and serotonin (green curve). (b) Fluorescence spectra of D-SWCNTs before (black curve) and after the addition of DA (orange curve) or serotonin (green curve), for D-SWCNTs with E_11_ < E_11_*, (c) E_11_ = E_11_*, and (d) E_11_ > E_11_*. The shaded areas represent the standard deviation in the respective measurements.

Control experiments were first conducted using pristine (6,5) enriched SC-SWCNTs, devoid of defects, incubated with DA and serotonin. Interestingly, the fluorescence intensity of pristine SC-SWCNTs exhibited a decrease of approximately 10% upon the addition of DA. Moreover, the fluorescence of SC-SWCNTs was quenched by about 28%, accompanied by a red shift of 4 nm upon adding serotonin (Figure 2a). Thus, it was clear that pristine SC-SWCNTs, in the absence of defects, evoked rather similar optical responses of the SWCNTs. Notably, these results align with a previous report, where SC-SWCNTs demonstrated lower final fluorescence intensity following serotonin addition compared to dopamine, along with a fluorescence shift in response to serotonin.^60^ On a similar note, instead of (6,5) enriched SC-SWCNTs, SWCNTs with mixed chirality dispersed with SC were incubated with DA and serotonin, and the resultant fluorescence spectra were recorded (Figure S2). Interestingly, DA did not lead to any discernible change in the fluorescence of the SC-SWCNTs, while serotonin led to a ∼18% decrease in the fluorescence intensity. The immediate next challenge was to determine if (6,5) enriched SWCNTs, which were similarly responsive to DA and serotonin, could exhibit differentiated optical responses upon the incorporation of defects.

Thus, in the next step, D-SWCNTs were incubated with DA and serotonin. In this set of experiments, the degree of defects in D-SWCNTs was controlled to a point where the fluorescence peak height associated with E_11_* surpassed that of E_11_. Intriguingly, upon the introduction of oxygen defects, DA increased the fluorescence intensity of D-SWCNTs by a factor of ∼6. On the contrary, serotonin led to a discernible decrease by a factor of ∼0.8 in the fluorescence intensity (Figure 2b). The diverse fluorescence responses of D-SWCNTs to structurally related neurotransmitters not only highlight the role of surface functionalities in nanoscale particles as a tool to alter their interactions with similar analytes but also allows for establishing them as effective probes for discriminating between these analytes.

In addition to the D-SWCNT sample in which the E_11_* peak was greater than E_11_ (Figure 2b), we prepared two additional dispersions of D-SWCNTs with distinct levels of defects characterized by different E_11_ to E_11_* ratios, having E_11_ nearly equal to E_11_* (Figure 2c), and E_11_ greater than E_11_* (Figure 2d), to test the effect of defect density. Upon exposure to DA, the increase in D-SWCNTs_E11>E11*_ fluorescence intensity was less than that of D-SWCNTs_E11<E11*_. On the other hand, the fluorescence intensity of both D-SWCNTs_E11>E11*_ and D-SWCNTs_E11<E11*_ was decreased upon interaction with serotonin. The largest fluorescence increase was observed in the case of D-SWCNTs_E11∼=E11*_. Nevertheless, the fluorescence of D-SWCNTs_E11=E11*_ was nearly nonresponsive to serotonin. To gain deeper insight into potential changes in the spectral pattern or possible shifts in the emission of D-SWCNTs upon the addition of DA and serotonin, the emission intensity was plotted on a logarithmic scale (Figure S3). The spectral patterns of D-SWCNTs were found to remain mostly unchanged following the addition of DA and serotonin, though a slight red shift was observed in D-SWCNTs with added serotonin, where the E_11_* intensity was greater than that of E_11_.

These results underscore the pivotal role of defect density within SWCNTs in eliciting divergent responses from DA and serotonin. Based on these findings, it can be inferred that, for optimal discrimination of DA and serotonin, D-SWCNTs with E_11_ < E_11_* would be an optimal choice. Therefore, D-SWCNTs_E11<E11_* was selected for further experimentation, as detailed in the subsequent sections.

Additionally, the UV–vis-NIR absorption spectra of D-SWCNTs were recorded both in the presence and absence of DA and serotonin (Figure S4). In the control samples without analytes, water was added to the D-SWCNT suspension to match the final volume of the analyte-containing samples, ensuring that any observed differences were not due to dilution effects. The peak at 988 nm in D-SWCNTs, associated with E_11_ transitions, demonstrated a decrease of approximately 7% upon adding DA. On the other hand, adding serotonin to D-SWCNTs resulted in a decrease of about 12% in the absorbance of the peak at 988 nm, accompanied by a bathochromic shift of approximately 10 nm. These findings, in line with previous studies showing that interactions between a chromophore and an analyte can alter the chromophore’s absorption spectrum, emphasize that the changes in the fluorescence of D-SWCNTs upon adding DA and serotonin could be attributed to interactions between D-SWCNTs and the respective analytes.^61^

### 2.3 Effect of Monoamine Compounds on the Fluorescence of D-SWCNTs

Among the various commonalities in the structural features of DA and serotonin, the presence of a single amine group is particularly notable. To further understand the role of amine groups in modulating the fluorescence of D-SWCNTs to DA and serotonin vis-à-vis pristine SWCNTs, D-SWCNTs with E_11_ < E_11_* were incubated with several other neurotransmitters, including histamine, glutamic acid, γ-aminobutyric acid (GABA), and glycine, alongside DA and serotonin. The rationale for choosing these neurotransmitters as model examples was based on their structure, having a single amine group. Additionally, some of these neurotransmitters have aliphatic chains, while others, such as histamine, contain aromatic rings (namely imidazole). The corresponding fluorescence spectra were recorded (Figure S5). Interestingly, glycine, GABA, and glutamic acid–the three aliphatic amines–caused an increase in the fluorescence intensity of D-SWCNTs by a factor of ∼1–2, while histamine, composed of an aromatic ring, led to an intensity increase by a factor of ∼3. It was interesting to observe that aromatic ring-containing neurotransmitters were found to elicit a greater increase in the fluorescence of D-SWNCTs as opposed to aliphatic chain-containing neurotransmitters. However, neurotransmitters with (mono) amine groups, regardless of the presence or absence of aromatic rings, were found to augment the fluorescence of D-SWCNTs. Notably, the increase in the fluorescence of D-SWCNTs upon interaction with these neurotransmitters was noticeably lower compared to DA, which enhanced the fluorescence of D-SWCNTs by a factor of approximately 6, despite having common structural features like amine groups, aromatic rings, and hydroxyl groups. On the other hand, serotonin, also a monoamine neurotransmitter containing an aromatic ring, was found to cause a pronounced decrease in the fluorescence of D-SWCNTs. This varied response among monoamine neurotransmitters prompted further investigation into the mechanism governing the fluorescence modulation of D-SWCNTs by dopamine and serotonin.

Additionally, we investigated the effect of other monoamine-based molecules, not categorized as neurotransmitters, on the fluorescence response of D-SWCNTs. To this end, levodopa (L-DOPA), ortho-aminophenol (OAP), L-tryptophan, and l-serine were incubated with D-SWCNTs, and the corresponding fluorescence spectra were recorded (Figure S6). Intriguingly, these monoamine-based molecules were observed to exert a slight (∼15%) enhancement in the fluorescence intensity of D-SWCNTs, particularly in the E_11_* peak at 1120 nm, which is attributed to the defects. This result further highlights that while amine-containing molecules can increase the fluorescence of defect-incorporated SWCNTs, the most prominent increase is caused by DA. In contrast, serotonin, which also contains a monoamine group, was found to distinctly decrease the fluorescence of the D-SWCNTs.

### 2.4 Calibration of D-SWCNTs for DA and Serotonin

**Figure 3:**
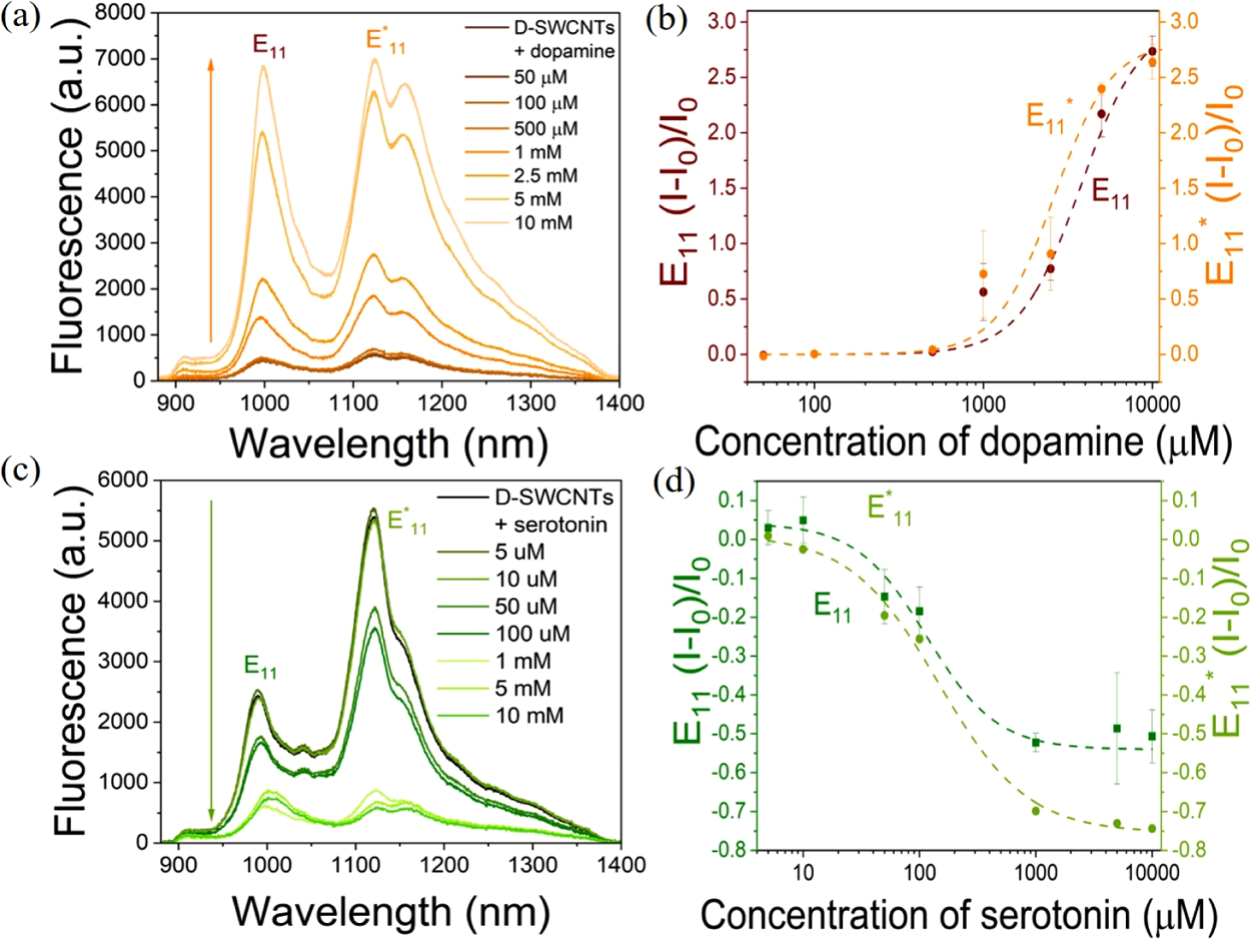
(a) Fluorescence spectra of D-SWCNTs before and after the addition of varying concentrations of DA. (b) Normalized fluorescence response of E_11_ (brown) and E_11_* (orange) peaks of D-SWCNTs toward varying concentrations of DA. (c) Fluorescence spectra of D-SWCNTs before and after the addition of varying concentrations of serotonin. (d) Normalized fluorescence response of E_11_ (dark green) and E_11_* (light green) peaks of D-SWCNTs toward varying concentrations of serotonin.

Having confirmed that the introduction of oxygen defects in SC-SWCNTs results in divergent effects of DA and serotonin on the fluorescence of the D-SWCNTs, an outcome not achievable without defect incorporation, we aimed to investigate the change in the fluorescence of D-SWCNTs upon the addition of varying concentrations of DA and serotonin. In this pursuit, the fluorescence intensities of the peaks at 988 and 1120 nm, attributed to E_11_ and E_11_* transitions, respectively, were observed to increase upon the addition of increasing concentrations of DA (Figure 3a). The normalized fluorescence responses of both E_11_ and E_11_* peaks were fitted using a Hill equation (Figure 3b, Table S1). The obtained dissociation constant *K*_d_ values from the fitting of E_11_ and E_11_* were determined to be 73.6 ± 8.8 and 51 ± 9.5 μM, respectively. The limit of detection (LOD) for DA by D-SWCNTs was calculated to be 3.7 ± 0.68 μM.

On the other hand, upon adding varying concentrations of serotonin to D-SWCNTs, the fluorescence intensities of the peaks at 988 and 1120 nm decreased (Figure 3c). In this case, the fluorescence response of the E_11_ and E_11_* peaks could also be fitted with a Hill equation (Figure 3d, Table S1). The *K*_d_ values calculated from the fitting of E_11_ and E_11_* peaks were found to be 2.6 ± 0.6 and 2.8 ± 0.5 μM, respectively. The LOD of serotonin was calculated to be 0.25 ± 0.027 μM. The obtained LODs for DA and serotonin by D-SWCNTs are comparable to those obtained by other techniques, including colorimetry,^62^ conductivity,^63^ cyclic voltammetry,^64^ and visible range fluorometry.^65^

### 2.5 Underlying Mechanism Governing the Fluorescence Intensity Enhancement of D-SWCNTs by DA

Having established the concentration-dependent divergent effects of DA and serotonin on the fluorescence of D-SWCNTs while also confirming the indispensable role of defects in achieving these effects, we aimed to understand the mechanism underlying the enhancement in the fluorescence intensity of D-SWCNTs upon adding DA.

The enhancement in the fluorescence intensity of pristine SWCNTs without defects upon interaction with DA is commonly attributed to the removal of reactive oxygen species (ROS).^53^ DA, being a redox-active molecule, can scavenge oxygen radicals. Additionally, it is well-established that the fluorescence of SWCNTs can be effectively quenched in the presence of ROS.^53^ Therefore, the removal of ROS due to their scavenging by DA is likely to increase the fluorescence intensity of the SWCNTs. However, in our study, the ROS of concern is singlet oxygen species, which have limited lifetimes in water, on the order of microseconds, suggesting that the effect of ROS would be minimal.^66^ Nevertheless, to investigate if the ROS-based mechanism of fluorescence enhancement applies to defect-induced emission of SWCNTs as well, we tested this possibility with D-SWCNTs. For this, we incubated pristine and D-SWCNTs with mannitol, a well-known radical scavenger. However, while the fluorescence of pristine SWCNTs remained practically unaltered, the fluorescence of D-SWCNTs showed a slight increase following interaction with mannitol, suggesting that the scavenging of oxygen radicals could not fully explain the enhancement in the fluorescence intensity of D-SWCNTs by DA (Figure S7). Furthermore, to rule out the effect of Na^+^ and ClO^–^ ions in enhancing the fluorescence of D-SWCNTs upon the addition of DA, we added DA to D-SWCNTs after removing NaClO via dialysis. Interestingly, even in the absence of NaClO, we still observed a fluorescence increase in D-SWCNTs following the addition of DA. This confirmed that NaClO did not play a role in the mechanism driving the fluorescence enhancement of D-SWCNTs upon DA addition (Figure S8).

Another plausible mechanism frequently invoked to elucidate the phenomenon of fluorescence enhancement in various nanoscale particles is that of surface passivation.^67−70^ Surface passivation is known to enhance the fluorescence of nanoparticles by reducing nonradiative decay, a process that competes with radiative channels, thereby increasing the fluorescence quantum yield.^69^ Passivating molecules on the surface can limit the access of molecules that cause fluorescence quenching, preventing solvent relaxation and spatial proximity of other quenchers. Additionally, surface defects may induce nonradiative states, and passivation can mitigate these effects, resulting in a substantial enhancement in the fluorescence of nanoparticles.^71^ We postulated that surface passivation might be a conceivable mechanism for the fluorescence enhancement of D-SWCNTs induced by DA. This speculation is based on the known propensity of DA to readily generate PDA, especially in an oxidative environment, which could potentially passivate the surface of D-SWCNTs, resulting in an augmented fluorescence signal.^72^ However, in our case, owing to the potential ability of the formed PDA to physically adsorb onto the surface of SWCNTs, we use the term “adsorption” in our subsequent discussion.

To further validate the potential formation of PDA from DA in the presence of D-SWCNTs, we initially examined the feasibility of PDA generation. The confirmation of PDA formation was obtained through electrospray ionization mass spectrometry (ESI-MS) analysis (Figure S9a), revealing a major peak at 227 Da,^73^ corresponding to the mass of a fragment of PDA. Further, additional peaks were observed due to the fragmentation of PDA, which have been assigned to specific chemical formulas in Table S2. In contrast, the ESI-MS spectrum of DA-added pristine SWCNTs showed major peaks at 154 Da (DA monomer) and 431 Da (sodium cholate), with no signature peak of PDA observed (Figure S9b). This also highlights the essential role of oxygen defects in mediating the transformation of DA to PDA and subsequent enhancement of D-SWCNT fluorescence upon DA addition, an effect not observed in SWCNTs lacking oxygen defects. To further substantiate the attribution of the 227 Da peak to PDA fragmentation, ESI-MS spectra of pristine and D-SWCNTs without the addition of DA were also acquired (Figure S9c–d). Interestingly, no discernible peak at 227 Da was observed in these spectra, affirming that the peak associated with PDA originated solely from monomeric DA. The PDA-mediated fluorescence enhancement in D-SWCNTs was consistent with the experimental observation that the extent of fluorescence enhancement upon the addition of DA after the removal of NaClO was lower than that observed without NaClO removal. This difference may be attributed to the pH of the dispersion, which was approximately 9 prior to NaClO removal, due to the hydrolysis of OCl^–^ to OH^–^, thus creating a basic environment. Under these conditions, the formation of PDA is known to be facilitated. Consequently, after the removal of OCl^–^ and the subsequent pH drop, the feasibility of PDA formation likely decreased, leading to limited PDA formation and, therefore, a reduced increase in the fluorescence of D-SWCNTs following DA addition. To further verify the role of PDA in facilitating surface adsorption and enhancing the fluorescence of D-SWCNTs upon DA addition, we examined the effect of DA addition at an acidic pH, where PDA formation is typically inhibited. Interestingly, under acidic conditions, the fluorescence intensity of D-SWCNTs decreased following DA addition (Figure S10), contrary to the increase noted at basic pH. This finding substantiates that when PDA formation is restricted, the fluorescence enhancement of D-SWCNTs upon DA addition does not occur, thereby further validating the role of PDA formation and its subsequent adsorption on D-SWCNTs as a potential mechanism underlying the observed fluorescence enhancement. To gain insights into the formation of PDA even after the removal of NaClO, we employed UV–vis absorption spectroscopy, where PDA formation is characterized by an absorption shoulder around 450 nm.^74^ We acquired absorption spectra of D-SWCNTs after NaClO removal, both with and without the addition of DA, along with the absorption spectrum of dopamine alone. Notably, neither D-SWCNTs without DA nor dopamine alone exhibited a distinct absorption shoulder at 450 nm.^74^ In contrast, the D-SWCNTs (post-NaClO removal) with DA displayed a clear absorption feature (Figure S11), indicating the formation of PDA.

**Figure 4:**
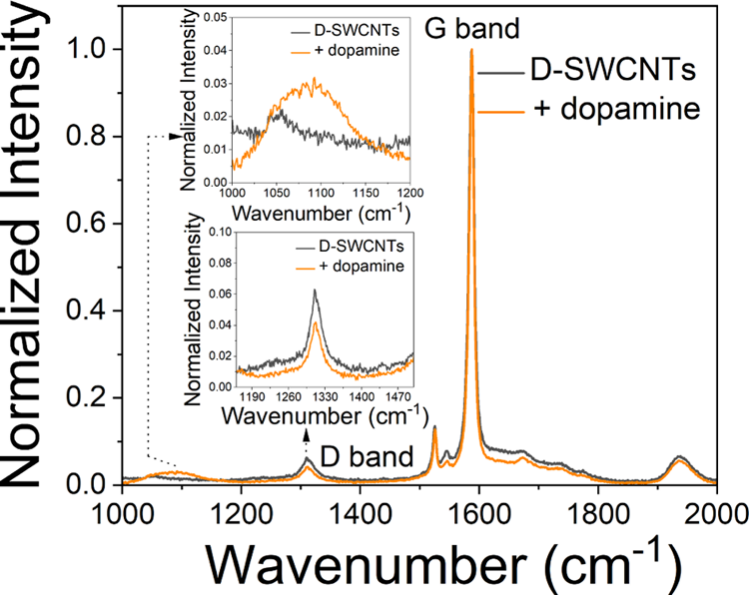
Raman spectra of D-SWCNTs before (black) and after the addition of DA (orange).

Having established the formation of PDA, our subsequent objective was to further substantiate the role of oxygen defects in the conversion of DA to PDA. It is worth-mentioning here that the essential role of oxygen defects in converting DA to PDA, and thereby causing an enhancement in the fluorescence of D-SWCNTs, seemed rather intuitive. This intuition was supported by existing literature, which reports that the introduction of aryl defects into SWCNTs, which do not promote the conversion of DA to PDA, results in a decrease in fluorescence upon the addition of DA.^46^ This suggests that oxygen defects could be crucial for the observed fluorescence enhancement in the presence of DA, as they likely facilitate the transformation of DA to PDA in an oxidative environment. However, as the reported effects of DA on aryl defect-induced SWCNTs were not studied with SC-coated SWCNTs, a direct comparison with our results remains limited. Thus, to verify this, we utilized Raman spectroscopy to scrutinize potential changes in the D band associated with defects upon adding DA. The Raman spectra of D-SWCNTs were recorded before and after the introduction of DA (Figure 4). Intriguingly, the intensity of the D band decreased upon the addition of DA, providing clear evidence of a chemical interaction between the defects within D-SWCNTs and DA. Furthermore, upon the addition of DA to D-SWCNTs, a peak at 1089 cm^–1^ emerged, typically originating from pentagonal defects in SWCNTs.^75^ Thus, in our case, it might be possible that the PDA molecules formed due to oxygen defects mediated conversion of DA might have been adsorbed onto the surface of SWCNTs, leading to some distortion in the hexagonal units. This adsorption could have generated localized strain in SWNCTs, causing carbon atoms to shift positions to relieve this tension. As a result, some hexagonal rings may have converted to nonhexagonal structures, potentially forming pentagonal units, as suggested by the emergence of the peak at 1089 cm^–1^. These additional pieces of evidence support the ubiquitous role of oxygen defects in the transformation of DA to PDA and the subsequent adsorption of PDA onto the surface of SWNCTs, ultimately leading to an enhancement in the fluorescence intensity of D-SWCNTs. Further, the validity of our claim regarding the adsorption of PDA onto the surface of D-SWCNTs leading to surface adsorption was further supported by normalizing the fluorescence spectra of D-SWCNTs before and after the addition of dopamine. After normalizing the spectra to the E_11_ peak, we observed a decrease in the intensity of the E_11_* peak (Figure S12). This indicates that while the addition of dopamine results in an overall increase in the fluorescence of D-SWCNTs, the relative contribution of the defects to the emitted fluorescence has decreased following the adsorption of PDA.

**Scheme 2:**
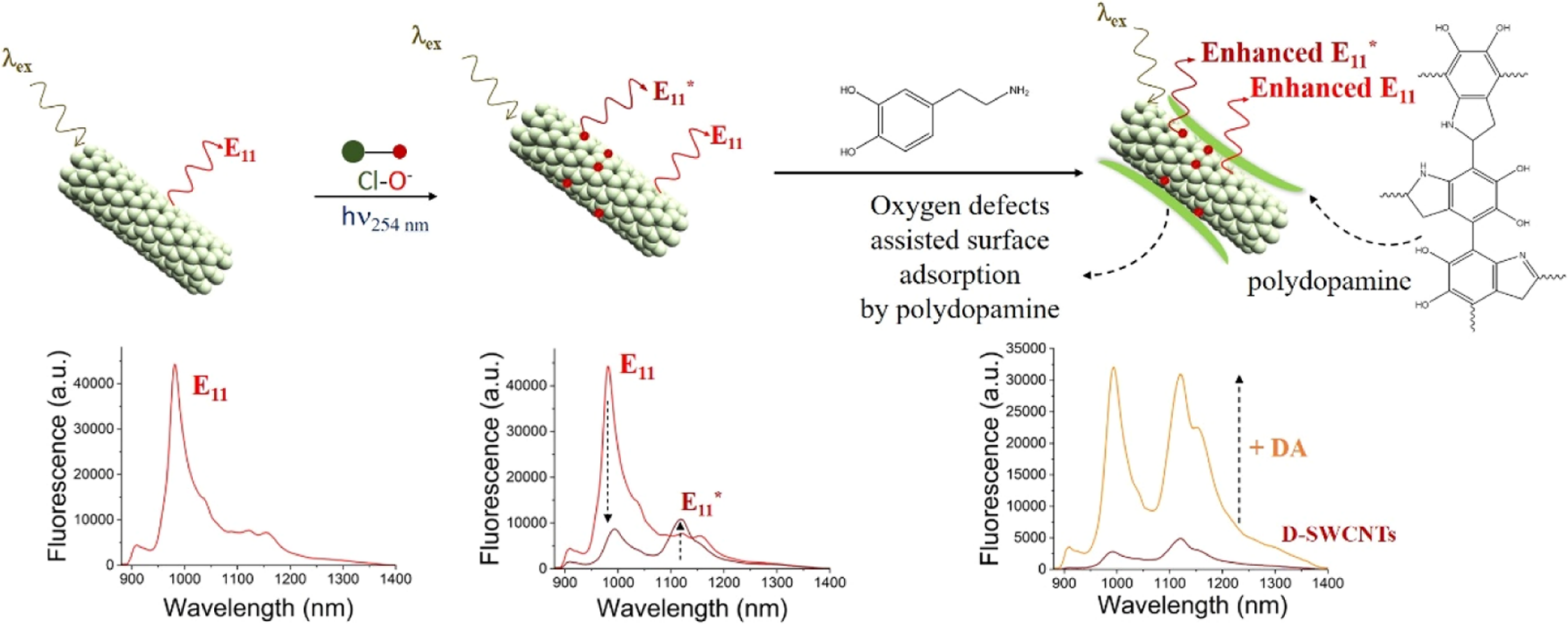
Schematic Illustration of Oxygen-defect Introduction Into SWCNT by Exposure to NaClO Under UV light (254 nm), Resulting in the Appearance of A New Peak at 1120 nm, Attributed to E_11_* Transitions and an Overall Decrease in the Fluorescence Intensity of D-SWCNTs Compared to Pristine SWCNTs Subsequently, the introduction of DA results in increased fluorescence intensity of the D-SWCNTs through the proposed mechanism of surface adsorption by PDA.

A schematic illustrating the plausible surface adsorption of D-SWCNTs by DA and the subsequent enhancement in the fluorescence of D-SWCNTs is shown in Scheme 2.

### 2.6 Underlying Mechanism Governing the Fluorescence Intensity Decrease of D-SWCNTs by Serotonin

In the subsequent phase, we aimed to unravel the plausible mechanism through which the fluorescence of D-SWCNTs was quenched upon the addition of serotonin. One potential scenario could involve a direct interaction between the defects present within the SWCNTs and serotonin, resulting in fluorescence modulation. However, Raman spectra of D-SWCNTs before and after adding serotonin showed no noticeable change in the D band of D-SWCNTs, associated with defects, upon the addition of serotonin (Figure S13). This suggests that the fluorescence quenching of D-SWCNTs by serotonin did not result from a direct chemical interaction between the defects and serotonin.

On a different note, a noteworthy observation was that in the absence of defects, serotonin quenched the fluorescence of pristine SC-SWCNTs by approximately 28% (Figure 2a). Interestingly, this extent of fluorescence reduction in SWCNTs by serotonin became more pronounced upon introducing defects, indicating that the defects further amplified the effect already present before their introduction. As discussed previously, the decrease in the fluorescence of pristine SC-SWCNTs upon adding serotonin was generally attributed to enhanced water accessibility due to changes in the surface coverage of SWCNTs.^60^ In this context, the increase in water accessibility is likely to be more pronounced in the presence of oxygen defects, potentially facilitated by hydrogen bonding between water molecules and oxygen defects within the SWCNTs. Consequently, the magnitude of the normalized fluorescence intensity decrease in D-SWCNTs upon adding serotonin might be more pronounced compared to pristine SWCNTs due to enhanced water accessibility facilitated by hydrogen bonding with the oxygen defects. This was further validated by the normalized spectra of D-SWCNTs before and after the addition of serotonin, where normalizing the spectra to the E_11_ peak revealed an independent decrease in the intensity of the defect-related peak (Figure S14). In this regard, electronic-to-vibrational energy transfer (EVET) to H_2_O molecules may also contribute to the observed decrease in fluorescence of D-SWNCTs upon the addition of serotonin.^46^

On the other hand, in line with PDA formation, as observed in the earlier section, we wanted to check the possibility of polyserotonin formation owing to the presence of oxygen defects in SWCNTs. To investigate the possibility of polyserotonin formation contributing to the decrease in fluorescence of D-SWCNTs following the addition of serotonin, we recorded the ESI-MS spectra of pristine and D-SWCNTs after serotonin addition (Figure S15a-b). However, neither spectrum exhibited peaks corresponding to fragments of polyserotonin. As evinced from Figure S15a–b, the peaks observed at 160 Da, 175 Da, and 431 could be ascribed to the fragmentation of serotonin (following the release of ammonia), parent serotonin, and SC, respectively. This negated the possibility of a contribution of polyserotonin to the observed decrease in the fluorescence of D-SWCNTs.

### 2.7 Divergent Response of D-SWCNTs to DA and Serotonin in Serum

**Figure 5:**
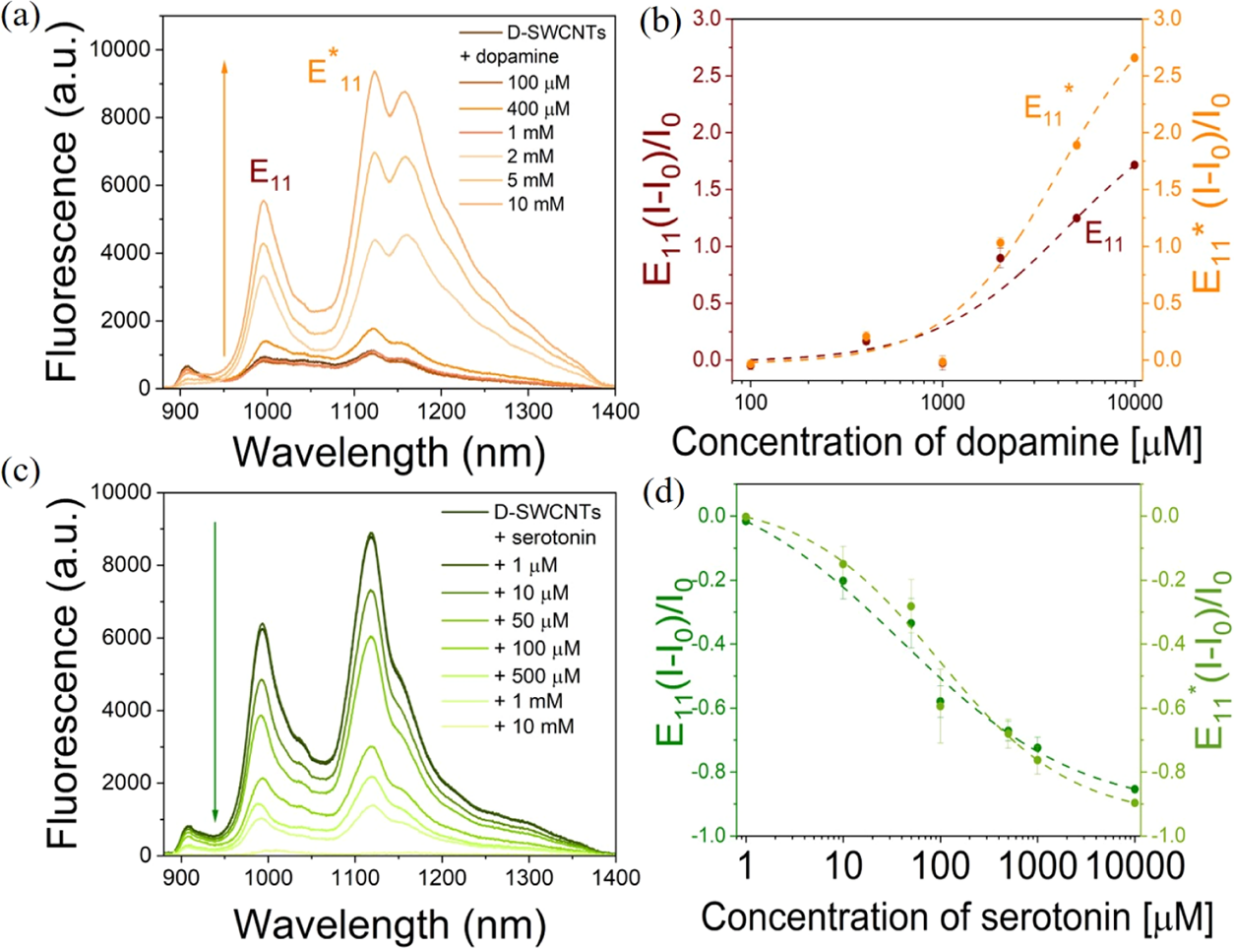
(a) Fluorescence spectra of D-SWCNTs before and after the addition of varying concentrations of DA in FBS. (b) Normalized fluorescence response of E_11_ (brown) and E_11_* (orange) peaks of D-SWCNTs toward varying concentrations of DA in FBS. (c) Fluorescence spectra of D-SWCNTs before and after the addition of varying concentrations of serotonin in FBS. (d) Normalized fluorescence response of E_11_ (dark green) and E_11_* (light green) peaks of D-SWCNTs toward varying concentrations of serotonin in FBS.

To validate the role of oxygen defects in the successful modulation of fluorescence response of SWCNTs DA and serotonin in real-world samples, we evaluated their performance in fetal bovine serum (FBS). Initially, we introduced varying concentrations of DA spiked into a 5% FBS solution, into a dispersion of D-SWCNTs, and recorded the resulting fluorescence. Similar to the response observed in aqueous solutions to DA (as depicted in Figure 3a), the fluorescence of D-SWCNT E_11_ and E_11_* transitions at 992 and 1121 nm, respectively, exhibited a concentration-dependent increase upon the addition of DA in a serum environment (Figure 5a). The fluorescence response of both E_11_ and E_11_* peaks, which demonstrated enhancement with increasing concentrations of DA, was normalized and fitted using a Hill eq (Figure 5b). The dissociation constant *K*_d_ values obtained from the fitting of E_11_ and E_11_* were determined to be 84 ± 30.0 and 77.6 ± 26.6 μM, respectively. Additionally, the LOD for DA by D-SWCNTs was calculated to be 372 ± 156 nM (Table S3).

Similarly, different concentrations of serotonin spiked into a 5% FBS solution were introduced with D-SWCNT suspension, and the resulting fluorescence was recorded. A consistent decrease in the fluorescence of D-SWCNTs was observed with the increasing concentration of serotonin, akin to the behavior observed in aqueous solutions of serotonin (Figure 5c). The fluorescence response of both the E_11_ and E_11_* peaks, which exhibited a decrease with increasing concentrations of serotonin, was normalized and fitted using a four-parameter logistic function with a zero baseline (Figure 5d). Notably, the calibration curve for serotonin in serum exhibited a better fit with the four-parameter logistic function with a zero baseline compared to the Hill fit. This is likely because the former more effectively accounts for both the lower and upper asymptotes. The concentrations of serotonin required for half-maximal response, as determined from fitting E_11_ and E_11_*, were found to be 0.8 ± 0.55 and 1.8 ± 0.52 μM, respectively. The limit of detection (LOD) was calculated to be 113 ± 27 nM (Table S4). Notably, the *K*_d_ and LOD values obtained in blood serum and aqueous medium are comparable, indicating the robustness of D-SWCNTs in eliciting divergent responses toward DA and serotonin in different interaction media. However, for dopamine, the LOD in serum was found to be an order of magnitude lower than in the aqueous system. While it is difficult to pinpoint an exact reason for this, one possible explanation is that oxidizing species present in serum may have promoted the oxidation of DA to PDA, which could have subsequently led to surface adsorption of the D-SWCNTs. This passivation might have enhanced the fluorescence of the nanotubes at lower dopamine concentrations, thus lowering the LOD in serum. It is also worth noting that the superior performance of SWCNT-based probes in serum compared to aqueous or buffer systems is well-documented in the literature.^24^ Additionally, it is important to highlight that the LODs for dopamine and serotonin in serum were comparable to their physiological concentrations, which generally fall within the submicromolar range.^76,77^ It is also important to acknowledge that DNA-suspended SWCNTs, as reported in the literature, typically achieve lower LODs for neurotransmitters, often in the nanomolar range.^53^ Nonetheless, as the primary aim of our study is not the precise detection of neurotransmitters but rather the development of defect-suspended SWCNTs to elicit distinct responses to chemically similar neurotransmitters, this difference in LOD does not pose a significant limitation to our objectives.

Conversely, pristine SC-SWCNTs, lacking defects, did not exhibit distinct changes in fluorescence when exposed to DA or serotonin spiked into 5% FBS (Figure S16). This underscores the crucial role of defects in distinguishing between DA and serotonin, even within a complex biological environment.

### 2.8 Divergent Optical Responses Following the Introduction of Oxygen Defects in SWCNTs Stabilized with the Lipid-PEG Corona

While SC-based corona for SWCNTs can potentially cause adverse effects in *in vivo* systems, for *in vitro* analyses, such as in complex biological fluids, no such adversity is expected.^60,78^ Nevertheless, to expand the applicability of our approach of using oxygen defects to achieve divergent responses of SWCNTs to structurally related analytes that would otherwise evoke similar responses, we introduced oxygen defects in SWCNTs suspended with lipid-PEG, namely, 1,2-distearoyl-*sn*-glycero-3-phosphoethanolamine-N-[carboxy(polyethylene glycol)-2000] DSPE-PEG. Lipid-PEG is well-recognized for its biocompatibility, and SWCNTs suspended with lipid-PEG have been extensively utilized in biological applications, demonstrating excellent biocompatibility.^78,79^

Specifically, (6,5) chirality-enriched SWCNTs were initially suspended with 2% SC. Subsequently, the SC molecules were replaced with DSPE-PEG via dialysis using standard protocols.^80^ The successful replacement of SC with DSPE-PEG was evinced from the bathochromic shift in the absorption peak of the E_11_ transition at 987 nm (Figure S17a).^80^ The DSPE-PEG-suspended SWCNTs were then introduced to oxygen defects in a manner similar to that of SC-SWCNTs. Notably, the introduction of oxygen defects resulted in the appearance of the E_11_* peak at 1120 nm, akin to what was observed in SC-SWCNTs with defects. However, unlike SC-SWCNTs, when identical concentrations of SWCNTs and NaClO and the same UV irradiation time were used, the intensity of the E_11_* peak reached only about half that of the E_11_ peak. This discrepancy may stem from the different coatings used for SWCNTs, DSPE-PEG in the present case versus SC in the standard case. To unequivocally confirm the incorporation of oxygen defects in DSPE-PEG-SWCNTs, Raman spectroscopy measurements were conducted. Notably, following treatment with NaClO and UV irradiation, a defect-characteristic peak emerged at 1312 cm^–1^ (Figure S17b), confirming the successful incorporation of oxygen defects in DSPE-PEG-SWCNTs. Under these conditions, DSPE-PEG-SWCNTs with defects-induced emission (DSPE-PEG-D-SWCNTs) exhibited a discernible increase in fluorescence when treated with dopamine (Figure S17c). Conversely, when treated with serotonin, DSPE-PEG-D-SWCNTs showed a notable decrease in fluorescence (Figure S17c). Importantly, pristine DSPE-PEG-SWCNTs, lacking defects, showed little distinction between dopamine and serotonin, as both resulted in decreased fluorescence of the SWCNTs—albeit to varying degrees (Figure S17d). These results are similar to those obtained using oxygen-incorporated SC-SWCNTs.

These results highlighted that the distinct optical responses of D-SWCNTs to DA and serotonin could be achieved with different corona phases involved in dispersing the D-SWCNTs, thereby underscoring the generality of the approach of introducing oxygen defects to evoke divergent responses from structurally related neurotransmitters, which otherwise produce similar responses from defect-free SWCNTs. Further, this study also lays the foundation for future applications of this concept in the facile discrimination of neurotransmitters *in vivo*. Another important future research direction could involve monitoring the reversibility of the interaction between structurally related neurotransmitters and D-SWCNTs, as well as fluorescence lifetime measurements, to extend our understanding of the photophysical properties of the D-SWCNT interaction with target analytes.

## 3 Conclusions

Tuning the optical response of NIR fluorescent SWCNTs toward structurally related molecules through incorporating oxygen defects, especially to achieve divergent responses from these similar molecules, remains a persistent challenge. In this study, oxygen defects were introduced in (6,5) enriched SWCNTs by a one-step reaction with NaClO under UV exposure. The successful incorporation of oxygen defects was validated through various analytical techniques, including UV–vis-NIR absorbance, NIR fluorescence, and Raman spectroscopy. Despite sharing similar functional groups, such as aromatic rings, −OH groups, and amine groups, DA and serotonin exhibited notably distinct effects on the fluorescence of D-SWCNTs, while pristine SWCNTs featured rather similar responses to DA and serotonin. Specifically, DA induced a substantial 6-fold increase in the fluorescence intensity of D-SWCNTs, while serotonin discernibly quenched the fluorescence of D-SWCNTs. Mechanistic investigations elucidated that the enhanced fluorescence of D-SWCNTs upon adding DA could be ascribed to surface adsorption by PDA formed through the polymerization of DA. Conversely, the decreased fluorescence of D-SWCNTs upon adding serotonin was linked to enhanced water accessibility resulting from changes in the surface coverage of SC-SWCNTs. Finally, DA and serotonin were observed to induce distinct responses in the fluorescence of D-SWCNTs, even in a serum environment.

This study is anticipated to deepen our understanding of how defects alter the surface chemistry of SWCNTs, leading to divergent optical responses from molecules that otherwise exhibit similar optical behavior with defect-free SWCNTs. Additionally, this research lays the groundwork for the discrimination of structurally related neurotransmitters, addressing a major challenge in the field of neurochemistry.

## 4 Experimental Section

### 4.1 Dispersion of SWCNTs with Sodium Cholate

To disperse (6,5) enriched CoMoCAT SWCNTs using sodium cholate (SC), 10 mg of SWCNTs were added to 20 mL of 2% SC. The mixture underwent 10 min of bath sonication followed by 30 min of tip sonication (12W) for two cycles, resulting in a SWCNT dispersion. Subsequently, the dispersion was ultracentrifuged at 41,300 rpm for 4 h, discarding the pellet of aggregated SWCNTs, and using the supernatant for further experiments. The concentration of the SC-SWCNTs was determined to be 163.8 mg L^–1^ through UV–vis-NIR absorption spectroscopy with an extinction coefficient of 0.036 L · mg^–1^ · cm^–1^ at 632 nm.

### 4.2 Absorption

Absorption spectra were obtained using a UV–vis-NIR spectrophotometer (Shimadzu UV-3600 Plus) covering the wavelength range of 300 to 1400 nm.

### 4.3 Introduction of Oxygen Defects in SC-SWCNTs

To induce oxygen defects in SC-SWCNTs, NaClO was utilized under UV irradiation. In brief, 10 μL of 11% NaClO was mixed with 990 μL of water, resulting in a 0.11% strength aqueous solution of NaClO. Subsequently, 500 μL of this solution was added to 500 μL of water, resulting in a 0.055% strength aqueous solution of NaClO. In a separate microcentrifuge tube, 20 μL of SC-SWCNTs (163.8 mg L^–1^) was diluted with 480 μL of water. This dilution likely led to a sparse coating of SC on the SWCNT surface.^60^ The dispersion of SC-SWCNTs was then added to 500 μL of the 0.055% NaClO solution alongside 100 μL of 0.11% NaClO solution. The sequential addition of NaClO was intended to prevent SWCNT aggregation and to control their oxidation, avoiding rapid or excessive defect formation. This dispersion was exposed to UV light at 254 nm. Reaction time-dependent fluorescence spectra of the resulting dispersions were obtained to confirm the incorporation of oxygen defects, as indicated by the emergence of a new peak at 1120 nm. D-SWCNTs_E11<E11*_, D-SWCNTs_E11=E11*_, and D-SWCNTs_E11>E11*_, shown in Figure 3 in the main text, were obtained following exposure of SC-SWCNTs added with said amount of NaClO to UV light for 4, 3, and 2 min, respectively.

### 4.4 Mass Spectrometry

Mass spectra were obtained using an LCMS Xevo–TQD instrument, and subsequent analysis utilized the Agilent 1260 system. The system incorporated a single quadrupole Mass Spectrometric Detector (MSD) equipped with a multimode ionization chamber capable of both Electrospray Ionization (ESI) and Atmospheric Pressure Chemical Ionization (APCI). Mass spectra were acquired in ESI-positive mode.

### 4.5 Fluorescence

Fluorescence emission spectra were recorded from samples in a 96-well plate positioned on the stage of an inverted microscope (Olympus IX73). A 730 nm continuous-wave laser (MDL-MD-730-1.5W, Changchun New Industries) served as the excitation source. The fluorescence emission spectra were resolved by a spectrograph (Spectra Pro HRS-300, Princeton Instruments) with a 500 μm slit-width and a grating density of 150 g mm^–1^. Recording of the fluorescence intensity spectrum was carried out using a 1D InGaAs array detector (PylonIR, Teledyne Princeton Instruments) with a 3 s exposure time. Excitation–emission maps were generated by sweeping the excitation wavelength range from 450 to 800 nm in 2 nm increments, facilitated by a supercontinuum white-light laser (NKT-photonics, Super-K Extreme).

### 4.6 Raman Spectroscopy

Raman spectroscopic measurements were conducted using a confocal micro-Raman (PL) spectrometer (LabRam HR Evolution). The samples were drop-cast onto a glass slide and excited with a 532 nm laser, while measurements were performed using a ×100 objective with a laser power of 100 mW. Spectra were collected in triplicate and are presented as the average of the three measurements.

### 4.7 Fluorometric Responses of D-SWCNTs to Neurotransmitters and Control Analytes

In a 96-well plate, 150 μL of as-prepared D-SWCNTs was added with 3 μL of a 10 mM (unless mentioned otherwise) aqueous solution of neurotransmitters and other analytes. The resulting dispersions were subjected to fluorescence spectrum measurement, with all spectra background-subtracted against blank water. Intensity values were obtained at the peak maximum for each spectrum, and the fluorescence responses were normalized to the initial fluorescence intensity, mitigating the influence of absolute fluorescence counts on quantitative results. The laser power utilized in the experiments ranged from 12 mW to 22 mW.

### 4.8 Divergent Responses from DA and Serotonin in Serum

DA and serotonin were dissolved in 1:20 diluted fetal bovine serum (FBS, Sigma) to attain a concentration of 10 mM of each. Subsequently, the DA and serotonin stock solutions were serially diluted with 1:20 FBS to achieve the desired concentrations for calibrating D-SWCNTs with respect to varying levels of DA and serotonin. For testing, 150 μL of D-SWCNTs was mixed with 3 μL of DA and serotonin at the desired concentrations (diluted in FBS), and the resulting changes in the fluorescence of the D-SWCNTs were recorded.

### 4.9 Suspension of SWCNTs with DSPE-PEG

SC-SWCNTs were exchanged with 1,2-distearoyl-*sn*-glycero-3-phosphoethanolamine-*N*-[carboxy(polyethylene glycol)-2000] (DSPE-PEG) using a dialysis method. Initially, a 5 mg mL^–1^ aqueous solution of DSPE-PEG was thoroughly sonicated to ensure full dissolution. Next, 40 mg L^–1^ of SC-SWCNTs was added to this solution to achieve a final concentration of 2 mg L^–1^ for DSPE-PEG. The resulting mixture underwent dialysis against water, over a period of 6 days with daily water changes. This purpose was to completely remove SC from the SWCNT surface, facilitating the adsorption of DSPE-PEG onto the SWCNTs.

### 4.10 Introduction of Defects in DSPE-PEG SWCNTs

To introduce oxygen defects in DSPE-PEG SWCNTs, 10 μL of 11% NaClO was mixed with 990 μL of water, resulting in a 0.11% strength aqueous solution of NaClO. Subsequently, 500 μL of this solution was added to 500 μL of water, resulting in a 0.055% strength aqueous solution of NaClO. In a separate microcentrifuge tube, 60 μL of SC-SWCNTs (53.6 mg L^–1^) was diluted with 440 μL of water. The dispersion of DSPE-PEG-SWCNTs was then added to 500 μL of the 0.055% NaClO solution alongside 100 μL of 0.11% NaClO solution. This dispersion was exposed to UV light at 254 nm for 5 min, resulting in the emergence of a new peak at 1120 nm and substantiating the incorporation of defects.

### 4.11 Statistical Analysis

Each sample’s fluorescence spectra were normalized to their initial fluorescence intensity. All fluorescence experiments were performed in triplicate, and the presented spectra are the mean of three independently acquired spectra. The fluorescence responses, illustrated in bar diagrams, represent averages of three independent measurements, with accompanying error bars denoting the standard deviation. Spectral data were plotted and analyzed using Origin software, while the excitation and emission profiles were graphed using MATLAB.

## Supporting Information Available

Spectroscopic characterization of D-SWCNTs, effects of dopamine and serotonin on the optical properties of SC-SWCNTs (HiPCO), the fluorescence of D-SWCNTs (logarithmic scale), spectroscopic characterization of lipid-PEG stabilized SWCNTs and D-SWCNTs, pristine SWCNTs in FBS, the effect of control molecules on the optical properties of D-SWCNTs, mass spectral analysis of D-SWCNTs with and without dopamine, dopamine and serotonin controls, the effect of dopamine on the optical properties of D-SWCNTs at acidic pH, polydopamine formation following NaClO removal, Raman spectra of D-SWCNTs before and after serotonin addition, tables with fit parameters of the calibration curves and LOD values for dopamine and serotonin detection in water and serum, and characterization of molecular fragments of polydopamine (PDF)

## Notes

A preprint of this work was posted on ChemRxiv with the following details: Basu, S.; Hendler-Neumark, A.; Bisker, G. The Role of sp^3^ Defects in Eliciting Divergent Fluorescence Response of Single-Walled Carbon Nanotubes to Dopamine and Serotonin. **2024**, ChemRxiv, 10.26434/chemrxiv-2024–848r1, July 30, 2024.
