# Supplementary material for: Role of Oxygen Defects in Eliciting a Divergent Fluorescence Response of Single-Walled Carbon Nanotubes to Dopamine and Serotonin
Source: ACS Nano. 2024 Dec 5;18(50):34134–46. doi: 10.1021/acsnano.4c10360 (PMC11656842; doi:10.1021/acsnano.4c10360)
Supplement: Supplementary file 1 — nn4c10360_si_001.pdf [file nn4c10360_si_001.pdf]

Supporting Information

The Role of Oxygen Defects in Eliciting Divergent Fluorescence Response of Single-Walled Carbon Nanotubes to Dopamine and Serotonin

Srestha Basu,[a] Adi Hendler-Neumark,[a] and Gili Bisker*[a,b,c,d]

[a] Department of Biomedical Engineering, Faculty of Engineering, Tel Aviv University, Tel Aviv 6997801, Israel

[b] Center for Physics and Chemistry of Living Systems, Tel Aviv University, Tel Aviv 6997801, Israel

[c] Center for Nanoscience and Nanotechnology, Tel Aviv University, Tel Aviv 6997801, Israel

[d] Center for Light-Matter Interaction, Tel Aviv University, Tel Aviv 6997801, Israel

Email: bisker@tauex.tau.ac.il

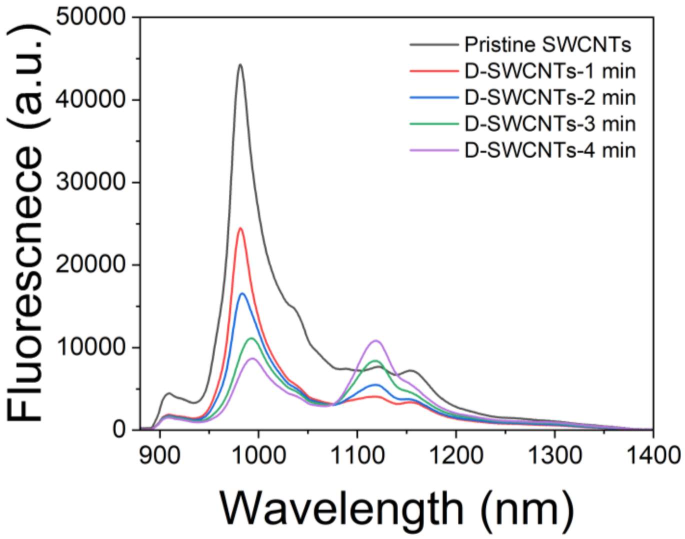

Figure S1: Unnormalized fluorescence spectra of SC-SWCNTs upon the addition of NaClO with increasing time of UV irradiation.

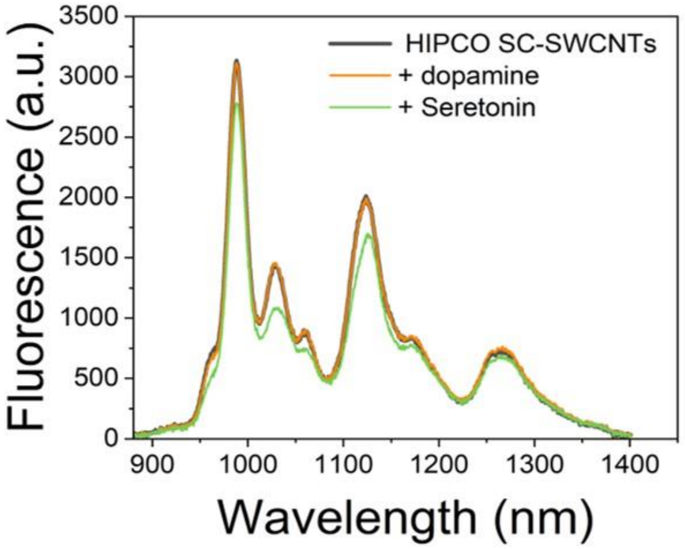

Figure S2: Fluorescence spectra of pristine SC-SWCNTs (black curve) and that following the addition of dopamine (orange curve) and serotonin (green curve).

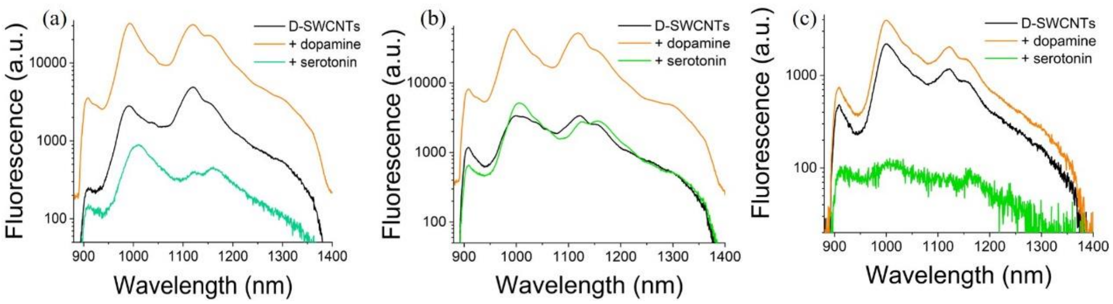

Figure S3: Fluorescence spectra of D-SWCNTs before (black curve) and after the addition of DA (orange curve) or serotonin (green curve), for D-SWCNTs with $E_{11} < E_{11}^*$, **(c)** $E_{11} = E_{11}^*$, and **(d)** $E_{11} > E_{11}^*$. The y-axes have been plotted in log scale.

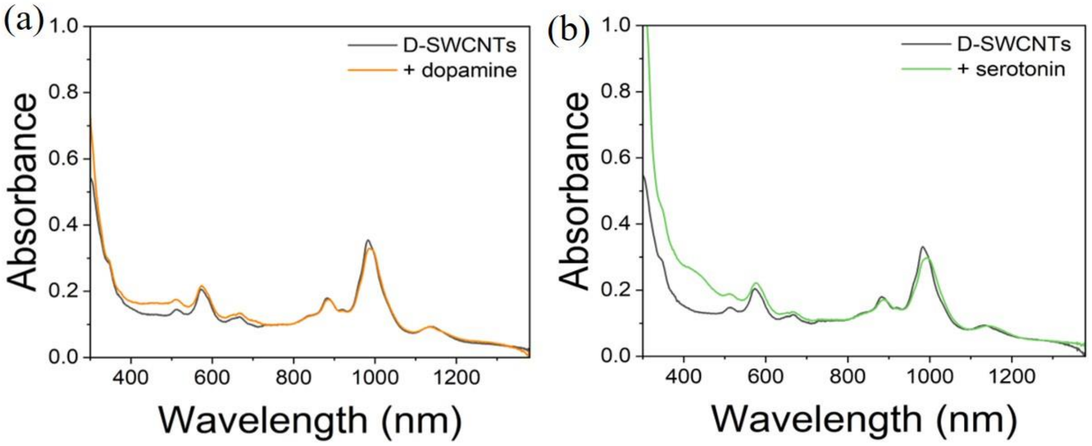

Figure S4: **(a)** UV-vis-NIR normalized absorption spectrum of D-SWCNTs (black curve) and that following the addition of dopamine (orange curve). **(b)** UV-vis-NIR absorption spectrum of D-SWCNTs (black curve) and that following the addition of serotonin (green curve).

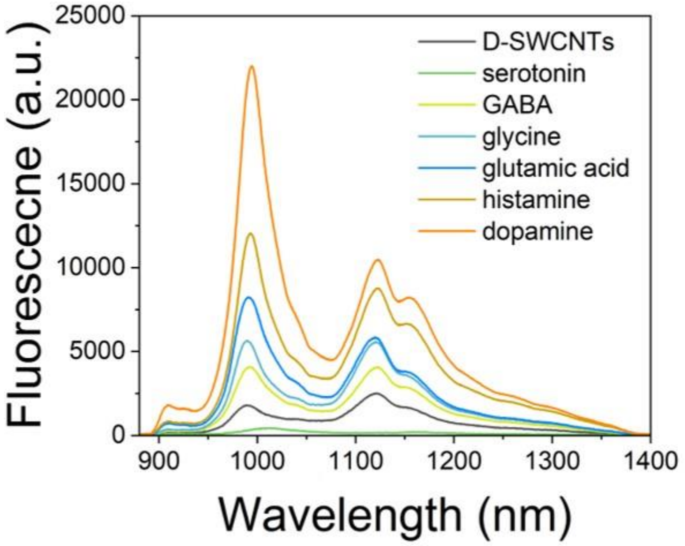

Figure S5: Fluorescence spectra of D-SWCNTs upon the interaction with various neurotransmitters, having common characteristics like aromatic rings, amine, and hydroxyl groups.

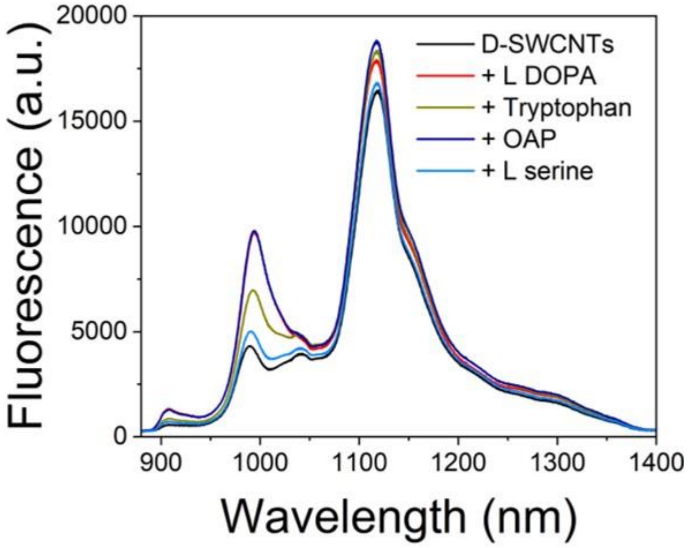

Figure S6: Fluorescence spectra of D-SWCNTs before and after the addition of various aromatic and aliphatic monoamines.

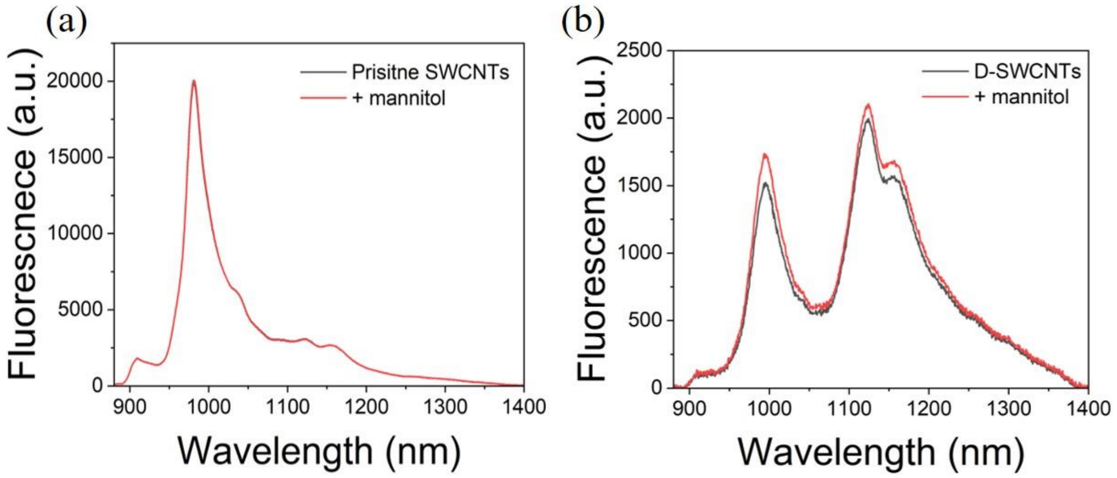

Figure S7: (a) Fluorescence spectra of pristine SWNCTs before (black) and after the addition of mannitol (red). **(b)** Fluorescence spectra of D-SWCNTs before (black) and after the addition of mannitol (red).

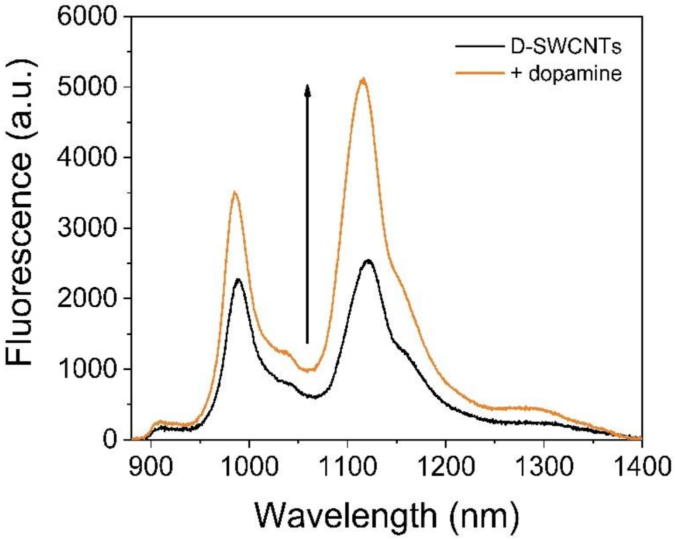

Figure S8: Fluorescence spectra of D-SWCNTs before (black) and after (orange) the addition of dopamine, following the removal of NaClO.

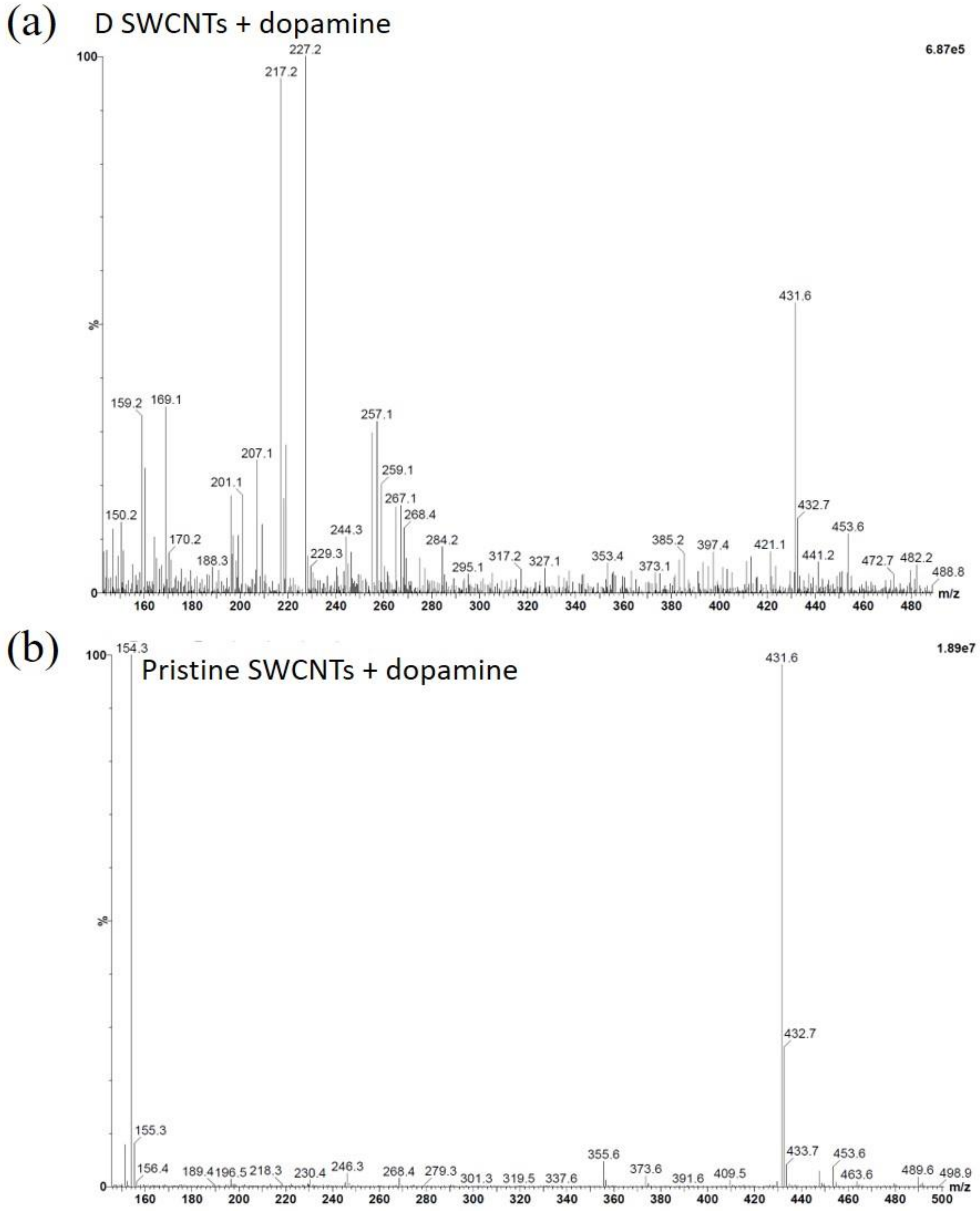
(a)
D SWCNTs + dopamine
6.87e5
100
%
0
227.2
217.2
431.6
159.2
169.1
257.1
207.1
259.1
201.1
267.1
268.4
150.2
432.7
453.6
244.3
284.2
170.2
385.2
397.4
421.1
229.3
353.4
188.3
317.2
327.1
441.2
472.7
482.2
295.1
373.1
488.8
160 180 200 220 240 260 280 300 320 340 360 380 400 420 440 460 480
m/z
(b)
Pristine SWCNTs + dopamine
1.89e7
100
%
0
154.3
431.6
432.7
155.3
355.6
433.7
453.6
156.4
189.4
196.5
218.3
230.4
246.3
268.4
279.3
301.3
319.5
337.6
373.6
391.6
409.5
463.6
489.6
498.9
160 180 200 220 240 260 280 300 320 340 360 380 400 420 440 460 480 500
m/z

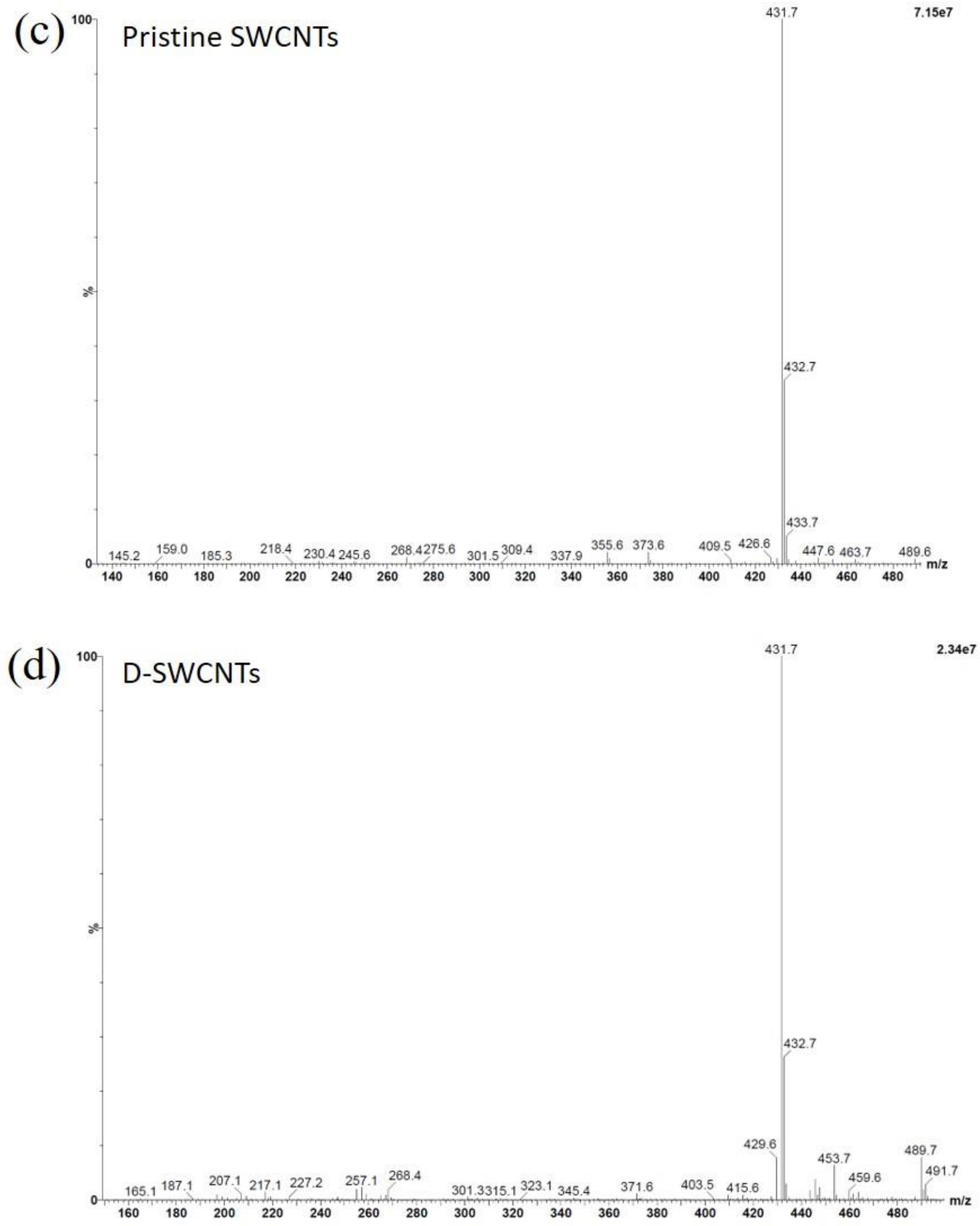

Figure S9: ESI-MS spectra of **(a)** D-SWCNTs, and **(b)** pristine SWCNTs added with dopamine. ESI-MS spectra of control (c) pristine SWCNTs, and (d) D-SWCNTs.

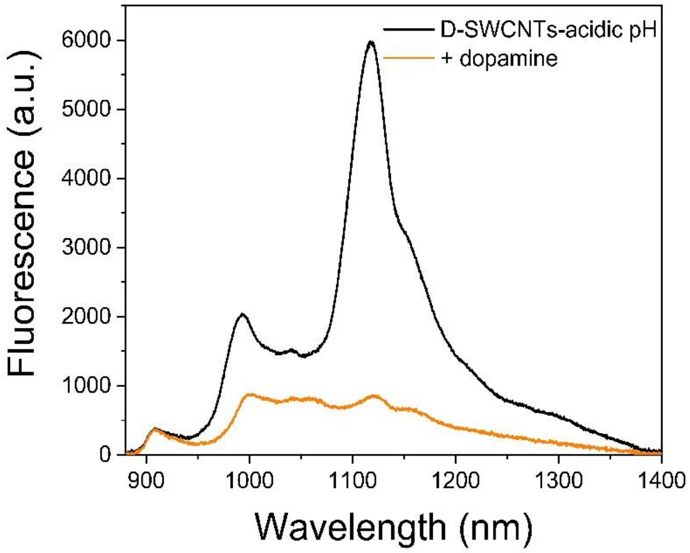

Figure S10: Fluorescence spectra of D-SWNCTs at acidic pH before (black) and after (orange) the addition of dopamine.

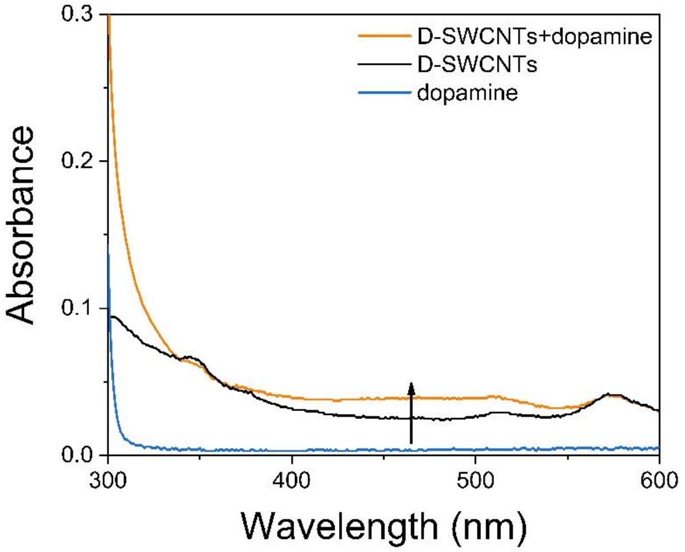

Figure S11: UV-vis absorption spectra of D-SWCNTs after NaClO removal, with dopamine addition (orange), without dopamine (black), and dopamine alone (blue).

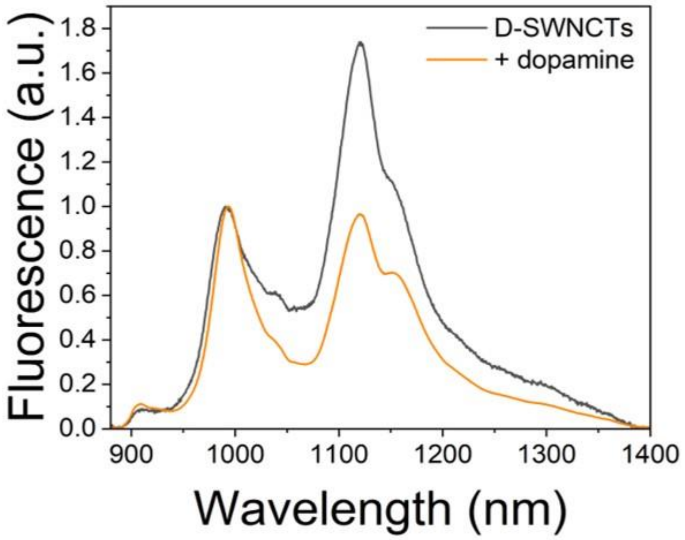

Figure S12: Normalized (to E_{11}) fluorescence spectra of D-SWNCTs before (black) and after (orange) the addition of dopamine.

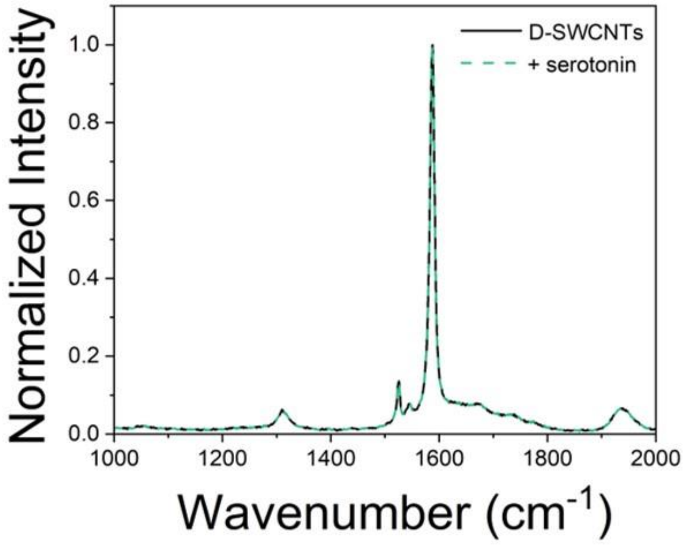

Figure S13: Raman spectra of D-SWCNTs before (black curve) and after the addition of serotonin (green curve).

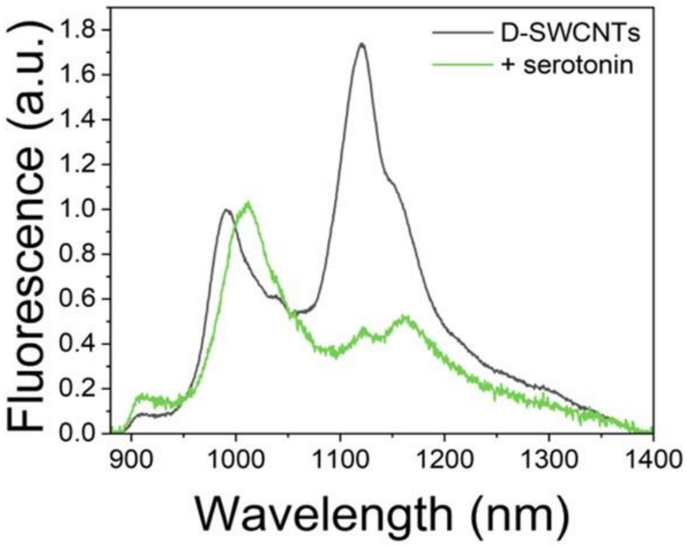

Figure S14: Normalized (to E_{11}) fluorescence spectra of D-SWNCTs before (black) and after (green) the addition of serotonin.

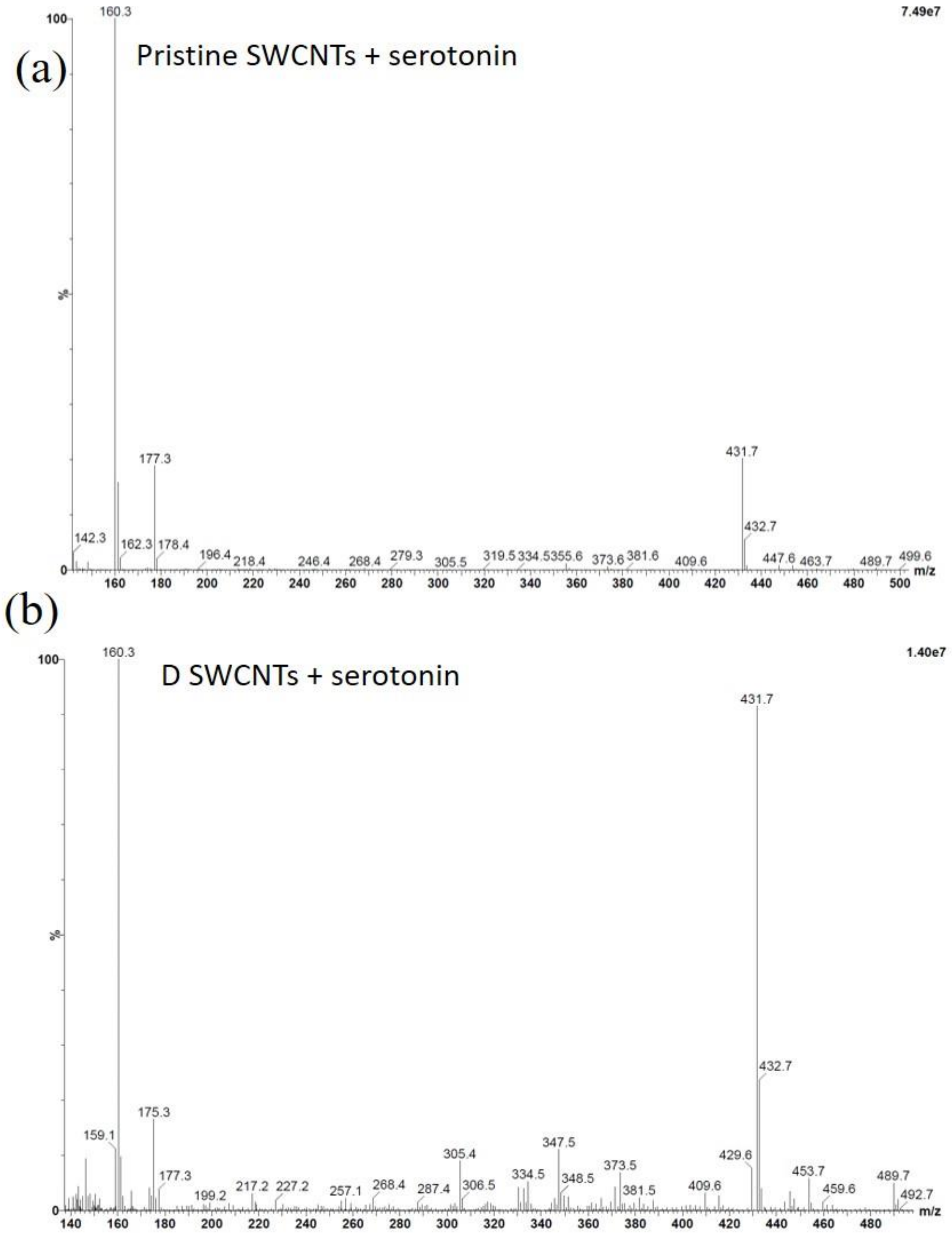

Figure S15: ESI-MS spectra of **(a)** pristine, and **(b)** D-SWCNTs added with serotonin.

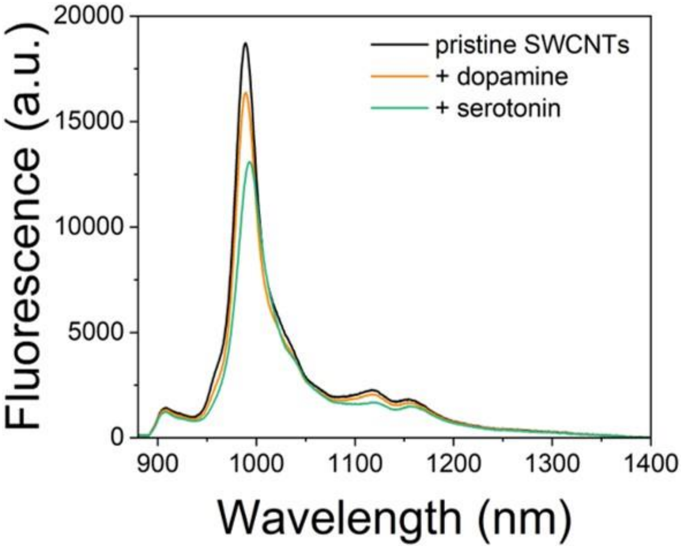

Figure S16: Fluorescence spectra of pristine SC-SWCNTs before (black curve) and after the addition of dopamine (orange curve) or serotonin (green curve), in FBS.

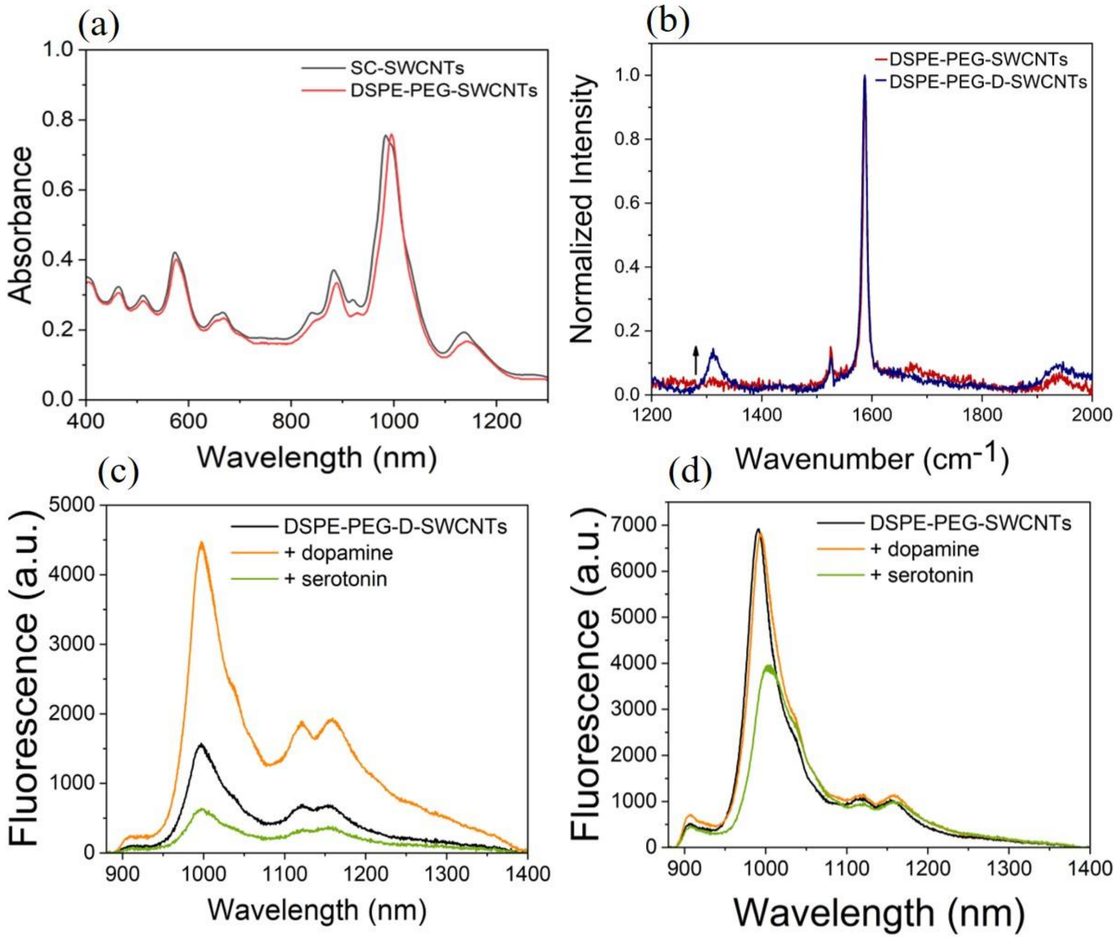

Figure S17. (a) Normalized absorption spectra of SC-SWCNTs suspension (black) and DSPE-PEG-SWCNT suspension (red). **(b)** Raman spectra of pristine DSPE-PEG-SWCNTs (red curve) and DSPE-PEG-D-SWCNTs (blue curve). **(c)** Fluorescence spectra of DSPE-PEG-D-SWCNTs before (black curve) and after the addition of dopamine (orange curve) and serotonin (green curve). **(d)** Fluorescence spectra of pristine DSPE-PEG-SWCNTs (black curve) and that following the addition of dopamine (orange curve) and serotonin (green curve).

Table S1: Hill fit parameters for DA in water. $\frac{I-I_0}{I_0} = \frac{\beta C^n}{K_d^n + C^n}$, *I* is the final fluorescence intensity, *I₀* is the initial fluorescence intensity, β is the proportion constant, K_d is the dissociation constant, *n* is the cooperativity factor, and *C* is the concentration of the analytes. LOD is the limit of detection.

**Sample**	$\boldsymbol{\beta}$		$\boldsymbol{K_d}$ **[µM]**		**LOD [µM]**	**Adjacent R^2**	
	E_{11}	E_{11}^*	E_{11}	E_{11}^*		E_{11}	E_{11}^*
D-SWCNTs + DA	3 ± 0.23	2.8 ± 0.26	73.6 ± 8.8	51 ± 9.5	3.7 ± 0.68	0.98	0.99
D-SWCNTs + serotonin	-0.54 ± 0.03	-0.77 ± 0.03	2.6 ± 0.6	2.8 ± 0.5	0.25±0.027	0.98	0.99

Table S2: Peak assignment of fragments of PDA observed in the ESI-MS spectrum

**Mass fragment**	**Chemical Formula**	**Chemical structures**
217.2	$[C_{22}H_{19}N_3O_6]^{2+}$	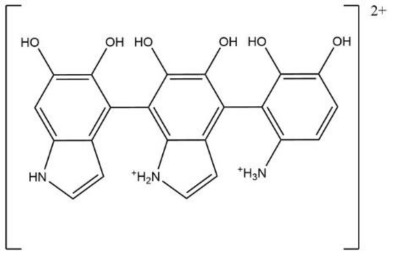
257.1	$[C_{15}H_{14}N_2O_2]$	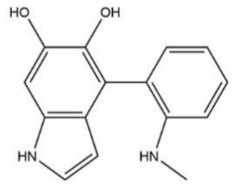

Table S3: Hill fit parameters for DA in FBS. $\frac{I-I_0}{I_0} = \frac{\beta C^n}{K_d^n + C^n}$, *I* is the final fluorescence intensity, *I₀* is the initial fluorescence intensity, β is the proportion constant, K_d is the dissociation constant, *n* is the cooperativity factor, and *C* is the concentration of the DA. LOD is the limit of detection.

**Sample**	$\boldsymbol{\beta}$		$\boldsymbol{K_d}$ **[µM]**		**LOD [nM]**	**Adjacent R^2**	
	E_{11}	E_{11}^*	E_{11}	E_{11}^*		E_{11}	E_{11}^*
D-SWCNTs + DA	2.3 ± 0.46	3.3 ± 0.65	84 ± 30.0	77.6± 26.6	372 ± 156	0.98	0.97

Table S4: Four parameters logistic fitting of serotonin with a zero baseline in FBS. $\frac{I-I_0}{I_0} = \frac{AB^n}{B^n+C^n}$, *I* is the final fluorescence intensity, I_0 is the initial fluorescence intensity, *A* is the proportion constant, *B* is the inflection point, and *C* is the concentration of serotonin. LOD is the limit of detection.

**Sample**	**A**		**B [µM]**		**LOD [nM]**	**Adjacent R^2**	
	**E_{11}**	**E_{11}^***	**E_{11}**	**E_{11}^***		**E_{11}**	**E_{11}^***
D-SWCNTs + serotonin	-0.92 ± 0.05	-0.94 ± 0.02	0.8 ± 0.55	1.8 ± 0.52	113 ± 27	0.999	0.999
